# The Transcriptional Activator LdtR from ‘*Candidatus* Liberibacter asiaticus’ Mediates Osmotic Stress Tolerance

**DOI:** 10.1371/journal.ppat.1004101

**Published:** 2014-04-24

**Authors:** Fernando A. Pagliai, Christopher L. Gardner, Lora Bojilova, Amanda Sarnegrim, Cheila Tamayo, Anastasia H. Potts, Max Teplitski, Svetlana Y. Folimonova, Claudio F. Gonzalez, Graciela L. Lorca

**Affiliations:** 1 Microbiology and Cell Science Department, Genetics Institute, Institute of Food and Agricultural Science, University of Florida, Gainesville, Florida, United States of America; 2 Soil and Water Science Department, Genetics Institute, Institute of Food and Agricultural Science, University of Florida, Gainesville, Florida, United States of America; 3 Department of Plant Pathology, Genetics Institute, Institute of Food and Agricultural Science, University of Florida, Gainesville, Florida, United States of America; Ohio State University, United States of America

## Abstract

The causal agent of Huanglongbing disease, ‘*Candidatus* Liberibacter asiaticus’, is a non-culturable, gram negative, phloem-limited α-proteobacterium. Current methods to control the spread of this disease are still limited to the removal and destruction of infected trees. In this study, we identified and characterized a regulon from ‘*Ca.* L. asiaticus’ involved in cell wall remodeling, that contains a member of the MarR family of transcriptional regulators (*ldtR*), and a predicted L,D-transpeptidase (*ldtP*). In *Sinorhizobium meliloti*, mutation of *ldtR* resulted in morphological changes (shortened rod-type phenotype) and reduced tolerance to osmotic stress. A biochemical approach was taken to identify small molecules that modulate LdtR activity. The LdtR ligands identified by thermal shift assays were validated using DNA binding methods. The biological impact of LdtR inactivation by the small molecules was then examined in *Sinorhizobium meliloti* and *Liberibacter crescens*, where a shortened-rod phenotype was induced by growth in presence of the ligands. A new method was also developed to examine the effects of small molecules on the viability of ‘*Ca.* Liberibacter asiaticus’, using shoots from HLB-infected orange trees. Decreased expression of *ldtR_Las_* and *ldtP_Las_* was observed in samples taken from HLB-infected shoots after 6 h of incubation with the LdtR ligands. These results provide strong proof of concept for the use of small molecules that target LdtR, as a potential treatment option for Huanglongbing disease.

## Author Summary

The rapid expansion of Huanglongbing disease (HLB) has caused a severe crisis in the citrus industry, with no solution visible in the near future. The causative agent, ‘*Candidatus* Liberibacter asiaticus’, is an unculturable bacterium under common laboratory conditions, which has made it difficult to gain understanding of this pathogen. Here we used a biochemical approach to identify new chemicals that could be used for the treatment of this devastating disease. These chemicals target a specific transcription factor (LdtR) in ‘*Ca.* Liberibacter asiaticus’. When bound to LdtR, the chemicals inactivate the protein, which disrupts a cell wall remodeling process that is critical for survival of the pathogen when exposed to osmotic stress (i.e. within the phloem of a citrus tree). Several model strains were used to confirm that the newly identified transcription factor (LdtR) and its regulated genes (*ldtR* and *ldtP*) confer tolerance to osmotic stress. The results presented in this study provide strong proof of concept for the use of small molecules that target LdtR, as a potential treatment option for Huanglongbing disease.

## Introduction

The rapid expansion of Huanglongbing (HLB; also known as “citrus greening”) disease caused a crisis in the citrus industry worldwide, with no solution visible in the near future. Experts estimate that without pro-active measures, the citrus industry in affected areas (like Florida) will be significantly reduced within 2–10 years. As such, it is critical to further our understanding of the metabolic and regulatory pathways in the causal agent ‘*Candidatus* Liberibacter asiaticus’ (‘*Ca.* L. asiaticus’), to facilitate the discovery of new means of prevention and/or treatment for HLB. Various treatment methods, including large scale field applications of penicillin and streptomycin, have been thoroughly examined and resulted in little success [1]. Although not applicable to field studies, thermotherapy (incubation of living plants in chambers at 40°C for 48 h) has been proposed for use in nurseries [2]. Despite all these efforts, current methods to control the spread of HLB are still limited to the removal and destruction of infected trees.

The causal agent of this devastating disease, ‘*Ca.* L. asiaticus’, is an unculturable bacterium. The inability to culture these species has greatly hindered progress toward the identification of therapeutic targets, and the development of viable treatment options. Furthermore, comparative genome analyses did not identify genes with predicted virulence functions (toxins), or specialized secretion systems (pathogenicity determinants) in the genome of ‘*Ca.* L. asiaticus’. These analyses did, however, provide valuable insight into the putative mechanisms of gene regulation.

Transcription factors, as defined by the Cluster of Orthologous Groups, constitute less than 2% of the ‘*Ca.* L. asiaticus’ genome, while in *S. meliloti*, another member of the *Rhizobiaceae* family, it comprises 6% of the genome. As a consequence, a small number of transcription factors may control several metabolic pathways. Therefore, we hypothesized that inactivation of a single transcription factor could result in pleiotropic effects, including decreased persistence within the host. CLIBASIA_01180 (renamed LdtR), is a homolog of the multidrug resistance regulator MarR. This regulator is encoded upstream of *CLIBASIA_01175*, a predicted L,D transpeptidase (renamed LdtP) involved in cell wall remodeling.

Peptidoglycan (PG) modifications have been observed in Gram-positive and Gram-negative bacteria, and often occur in response to environmental changes. The bacterial pathogens *Neisseria gonorrhoeae* and *Listeria monocytogenes* modify their PG residues to evade detection by the host immune system, and increase tolerance to stress [3], [4]. The PG structure consists of alternating N-acetylglucosamine (NAG) and β-(1-4)-N-acetylmuramic acid (NAM) residues. A peptide stem linked to the NAM residue mediates the cross-link to other units in the growing PG, forming a three-dimensional mesh-like architecture that confers structural strength and rigidity to the cell wall [5]. The PG of *A. tumefaciens* and *S. meliloti* is highly cross-linked (64%), with the muropeptide NAM-L-alanine, D-glutamic acid, DL-diaminopimelic acid, D-alanine being the most frequent [6].

The goal of this study was to characterize and assess the biological importance of LdtR and LdtP, and their role in the persistence of ‘*Ca.* L. asiaticus’ within citrus hosts. We used a biochemical approach to identify small molecules that modulate the expression and activity of the LdtR transcription factor. As ‘*Ca.* L. asiaticus’ is yet to be cultured, we used two of its closest culturable phylogenetic relatives, *Sinorhizobium meliloti* and *Liberibacter crescens*, as models to assess the biological role of LdtR and LdtP. We also developed a model using ‘*Ca.* L. asiaticus’ infected shoots, to validate LdtR as an effective target for the design of new therapeutics.

## Results

### LdtR binds to its own promoter region and to the *ldtP* promoter region

**Figure 1:**
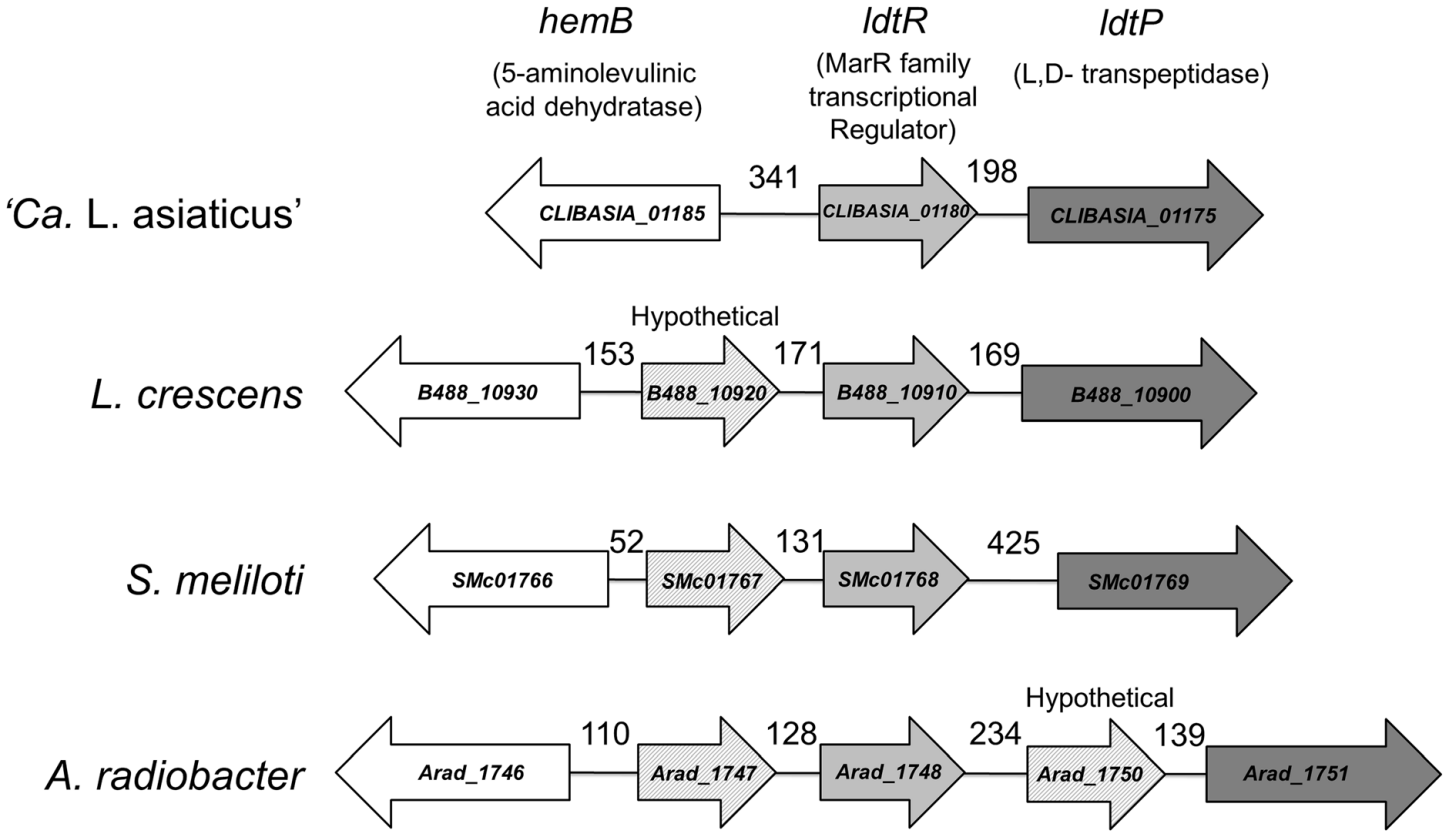
Genomic environment of *ldtR*. Homologs to *ldtR_Las_* are surrounded by similar genes in all analyzed members of the *Rhizobiaceae* family. The size of the intergenic region is indicated in each case. Homologs are depicted with identical colors. *CLIBASIA_01180* (gi|346722692), *CLIBASIA_01175* (gi|346722692), *CLIBASIA_01185* (gi|346722692); *B488_10930* (gi|431805346), *B488_10920* (gi|431805346), *B488_10910* (gi|431805346), *B488_10900* (gi|431805346); *SMc01766* (gi|15963753), *SMc01767* (gi|15963753), *SMc01768* (gi|15963753), *SMc01769* (gi|15963753); *Arad_1746* (gi|222084201), *Arad_1747* (gi|222084201), *Arad_1748* (gi|222084201), *Arad_1750* (gi|222084201), *Arad_1751* (gi|222084201).

The *ldtR* gene encodes the only MarR family member of transcriptional regulators in the genome of ‘*Ca.* L. asiaticus’ psy62 (*ldtR_Las_*). It shares high amino acid sequence identity to proteins found in all *Rhizobiaceae* family, including: ‘*Ca.* L. solanacearum’ CLso-ZC1 (89%), *Liberibacter crescens* BT-1 (73%), *Sinorhizobium meliloti* 1021 (70%), *Agrobacterium tumefaciens* F2 (74%), *A. radiobacter* K84 (71%), *Rhizobium leguminosarum* bv. *viciae* 3841 (71%), and *Hoeflea phototrophica* DFL-43 (65%). The genomic arrangement of *ldtR_Las_* was also similar to that of its orthologs (Fig. 1); however, none of these proteins has previously been characterized.

**Figure 2:**
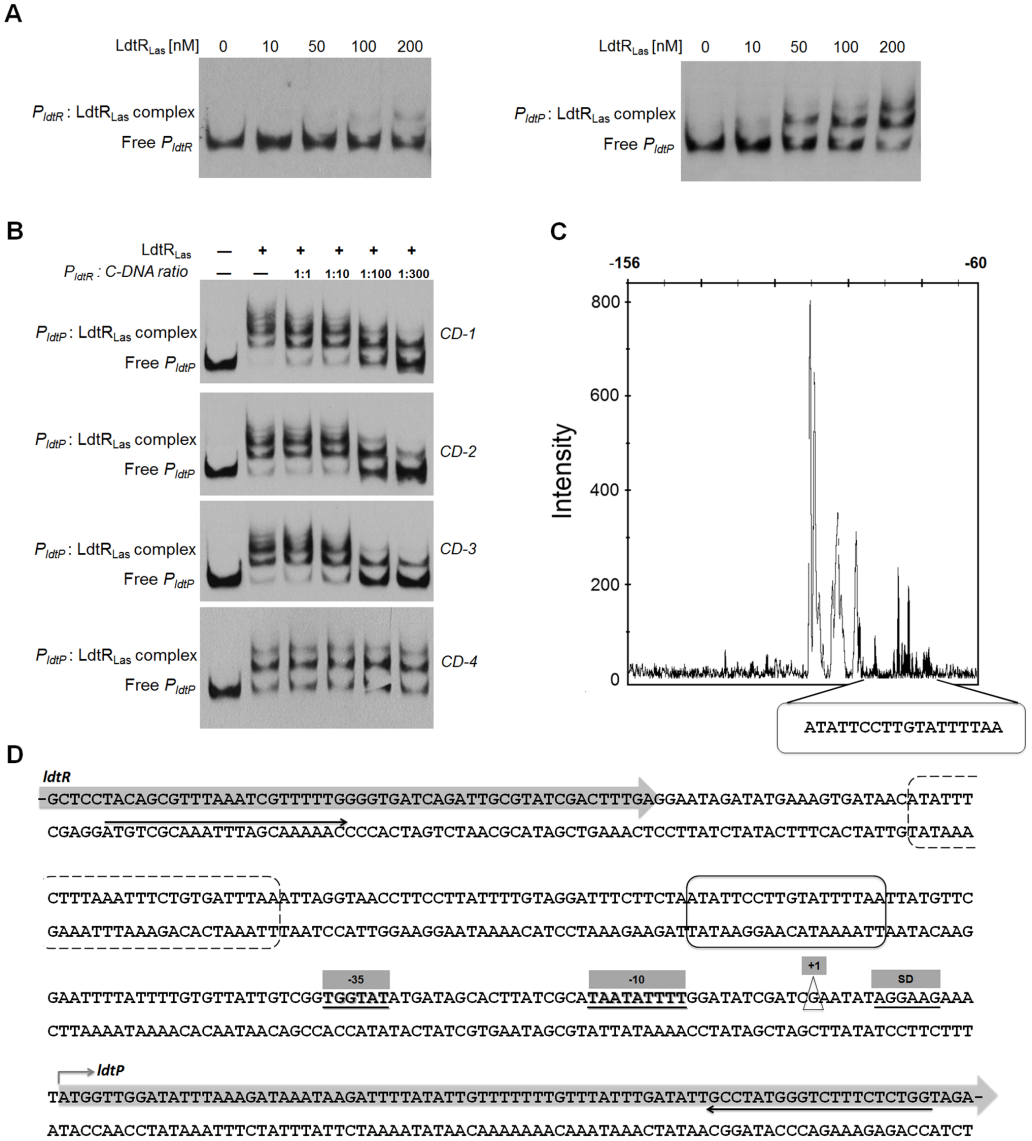
LdtR_Las_ binds to *P_ldtR_* and *P_ldtP_* of ‘*Ca.* L. asiaticus’. (A) EMSAs were conducted with increasing concentrations of LdtR_Las_, as indicated on top of each panel. No protein was added to the first lane. (B) Competition experiments. The biotin labeled *P_ldtP_* probe was incubated with 400 nM LdtR_Las_ and mixed with increasing concentrations of three different unlabeled double-stranded DNA fragments (*CD-1*, *CD-2*, *CD-3*, and *CD-4*). (C) Identification of LdtR_Las_ binding site in *ldtP* promoter (*P_ldtP_*). DNase I footprint electropherogram shows a fragment of the digested probe in absence (black) or presence (white) of LdtR_Las_, highlighting the protected region. LdtR_Las_ binding site is indicated with a circled box in panels C and D. (D) Characterization of *P_ldtP_*. The transcription start site (+1) was experimentally determined using 5′ RACE-PCR and it is depicted in a triangle. The predicted −10 and −35 boxes, as well as the Shine-Dalgarno sequence (SD) are underlined and highlighted in gray boxes. A putative second binding site for LdtR_Las_ is circled in a dashed box. Black arrows underneath the sequence denote the location of the primers used to generate the DNA probe for EMSA.

*ldtR_Las_* is encoded by the minus strand. *CLIBASIA_01185* is encoded 341 bp upstream of *ldtR_Las_*, on the plus strand. This gene encodes for a putative delta-aminolevulinic acid dehydratase (*hemB*) involved in tetrapyrrole biosynthesis [7]. Downstream of *ldtR_Las_*, on the minus strand, is *ldtP_Las_*, which contains both a YkuD L,D-transpeptidase domain (pfam03734) and a peptidoglycan binding domain (pfam01471), suggesting that it likely acts as an L,D-transpeptidase. Biotinylated probes were generated to contain the intergenic region of *CLIBASIA_01185* and *ldtR_Las_* (*P_ldtR_*: −395 to +47, positions are relative to *ldtR_Las_* translation start site), as well as the putative promoter region of *ldtP_Las_* (*P_ldtP_*: −248 to +79, relative to the *ldtP_Las_* translation start site). EMSA analysis of the interaction between LdtR_Las_ and *P_ldtR_* or *P_ldtP_*, revealed higher binding affinity for *P_ldtP_*, with 50% binding achieved at 100 nM (Fig. 2A). With increasing concentrations of LdtR_Las_, a higher molecular weight oligomer was also observed. Size exclusion chromatography indicated that LdtR_Las_ is a stable dimer in solution with an observed molecular weight of 39 kDa (Fig. S1). Taken together, these results suggest that there is either a second binding site within the *ldtP* promoter, or LdtR_Las_ may further oligomerize upon binding to DNA.

To confirm the location of LdtR binding, competitor experiments were conducted using unlabeled DNA probes (Table 1). The largest probe (*CD-1*) contains the whole sequence used in EMSA (from −248 to +79). Probe *CD-2* contains LdtR_Las_ binding site surrounded by promoter elements (−139 to +79). Probe *CD-3* was designed to contain only the protected site I identified by DNase I footprinting (−118 to −74), while probe *CD-4* does not contain the LdtR_Las_ binding site (−21 to +58). The addition of probe *CD-1* or *CD-2* resulted in a similar decrease in the intensity of the shifted bands (Fig. 2B). This effect was further enhanced in the presence of probe *CD-3*. No competition was observed with probe *CD-4*. These results indicate that LdtR_Las_ may have two binding sites within the *ldtP* the promoter.

**Table 1:** Primers used in this study.

Primer	Oligonucleotide sequence (5′→3′)	Target
**Protein purification**		
LdtR_Las__Fw^aa^	ttgtatttccagggcatgaacaataatatacaatcaaagataatttcg	‘*Ca.* L. asiaticus’
LdtR_Las__Rv^aa^	caagcttcgtcatcatcaaagtcgatacgcaatctgatcaccc	‘*Ca.* L. asiaticus’
LdtR_Smc__Fw^aa^	ttgtatttccagggcatgaacaccaagatgaag	*S. meliloti*
LdtR_Smc__Rv^aa^	caagcttcgtcatcattagaggcgatagaggattt	*S. meliloti*
**EMSA and DNase I footprint**		
PLdtR_Las__Fw^b^	tctcatacgtcgatgacaaca	‘*Ca.* L. asiaticus’
PLdtR_Las__Rv	gcactatccgtgtccgaaat	‘*Ca.* L. asiaticus’
PLdtP_Las__Fw^b^	ccagagaaagacccataggc	‘*Ca.* L. asiaticus’
PLdtP_Las__Rv	tacagcgtttaaatcgtttttg	‘*Ca.* L. asiaticus’
PLdtP_Las__fprint_*FAM*_Fw	ccagagaaagacccataggc	‘*Ca.* L. asiaticus’
PLdtP_Las__fprint_*VIC*_Rv	tacagcgtttaaatcgtttttg	‘*Ca.* L. asiaticus’
Competitor CD-1_Fw	tacagcgtttaaatcgtttttg	‘*Ca.* L. asiaticus’
Competitor CD-1_Rv	ccagagaaagacccataggc	‘*Ca.* L. asiaticus’
Competitor CD-2_Fw	ccttccttattttgtaggatttcttc	‘*Ca.* L. asiaticus’
Competitor CD-2_Rv	ccagagaaagacccataggc	‘*Ca.* L. asiaticus’
Competitor CD-3_Fw	tcttctaatattccttgtattttaattatgttcgaattttatttt	‘*Ca.* L. asiaticus’
Competitor CD-3_Rv	aaaataaaattcgaacataattaaaatacaaggaatattagaaga	‘*Ca.* L. asiaticus’
Competitor CD-4_Fw	cgatcgaatataggaagaaatatgg	‘*Ca.* L. asiaticus’
Competitor CD-4_Rv	atatcaaataaacaaaaaaacaata	‘*Ca.* L. asiaticus’
PCLIBASIA_01185_Fw^b^	tctcatacgtcgatgacaaca	‘*Ca.* L. asiaticus’
PCLIBASIA_01185_Rv	atcgcacatcgaatacgtca	‘*Ca.* L. asiaticus’
PLdtR_Smc__Fw	gttgctgatcgtgctccac	*S. meliloti*
PLdtR_Smc__Rv^b^	accacggatggtttcttcct	*S. meliloti*
PLdtP_Smc__Fw	ggcgggtttaccttcagtct	*S. meliloti*
PLdtP_Smc__Rv^b^	cgcgagaaagcatcaattc	*S. meliloti*
**Site-directed mutagenesis**		
pLdtP_M1_Fw	tttgtaggatttcttctaggattccttgtattttaatta	‘*Ca.* L. asiaticus’
pLdtP_M1_Rv	taattaaaatacaaggaatcctagaagaaatcctacaaa	‘*Ca.* L. asiaticus’
pLdtP_M2_Fw	gtaggatttcttctaataggccttgtattttaattatgt	‘*Ca.* L. asiaticus’
pLdtP_M2_Rv	acataattaaaatacaaggcctattagaagaaatcctac	‘*Ca.* L. asiaticus’
pLdtP_M3_Fw	ggatttcttctaatattccgggtattttaattatgttcg	‘*Ca.* L. asiaticus’
pLdtP_M3_Rv	cgaacataattaaaatacccggaatattagaagaaatcc	‘*Ca.* L. asiaticus’
pLdtP_M4_Fw	tttcttctaatattccttagattttaattatgttcgaat	‘*Ca.* L. asiaticus’
pLdtP_M4_Rv	attcgaacataattaaaatctaaggaatattagaagaaa	‘*Ca.* L. asiaticus’
pLdtP_M5_Fw	cttctaatattccttgtagtgtaattatgttcgaatttt	‘*Ca.* L. asiaticus’
pLdtP_M5_Rv	aaaattcgaacataattacactacaaggaatattagaag	‘*Ca.* L. asiaticus’
**5′RACE-PCR**		
LdtR_Las__RACE	agcactatccgtgtccgaaattatc	‘*Ca.* L. asiaticus’
LdtP_Las__RACE	ccagagaaagacccataggcaatatc	‘*Ca.* L. asiaticus’
LdtR_Lcr__RACE	tacggccttgacgttcaaat	*L. crescens*
LdtP_Lcr__RACE	gcgcaaaagaaggaactgac	*L. crescens*
Oligo_RACE	cgcgaattcctgtagaacgaacactaga	
Oligo_RACE_RNA	auaugcgcgaauuccuguagaacgaacacuagaagaaa	
**pDG1663 cloning**		
pDG1_HindIII_Fw^c^	gcgagaaagctttctcatacgtcgatgacaaca	‘*Ca.* L. asiaticus’
pDG2_BamHI_Rv^c^	ggagcgggatccagcactatccgtgtccgaaat	‘*Ca.* L. asiaticus’
pDG3_BamHI_Rv^c^	ggagcgggatcctcaaagtcgatacgcaatctg	‘*Ca.* L. asiaticus’
pDG4_HindIII_Fw^c^	gcgagaaagctttacagcgtttaaatcgtttttg	‘*Ca.* L. asiaticus’
pDG5_BamHI_Rv^c^	ggagcgggatcccagagaaagacccataggcaat	‘*Ca.* L. asiaticus’
pDG6_BamHI_Fw^c^	ggagcgggatcctctcatacgtcgatgacaaca	‘*Ca.* L. asiaticus’
pDG7_HindIII_Rv^c^	gcgagaaagctttcaaagtcgatacgcaatctg	‘*Ca.* L. asiaticus’
pDG8_HindIII_Rv^c^	gcgagaaagcttagcactatccgtgtccgaaat	‘*Ca.* L. asiaticus’
pDG9_seq_Fw	agcgccattcgccattcaggct	*B. subtilis*
pDG10_seq_Rv	tgcactatcaacacactcttaagtt	*B. subtilis*
Bs rpoB_Fw	ccgttgtttccagaaatcgt	*B. subtilis*
Bs rpoB_Rv	attacgttacggccaagtgc	*B. subtilis*
**pVMG cloning**		
LdtP_Smc_-1_AgeI_Fw^c^	cgaccggtatgcgctgaacgaaatc	*S. meliloti*
LdtP_Smc_-1_SpeI_Rv^c^	cgactagtcagggaatactcgccgatcac	*S. meliloti*
LdtP_Smc_-2_AgeI_Fw^c^	cgaccggttgtcctcgacgaagatcaact	*S. meliloti*
LdtP_Smc_-2_SpeI_Rv^c^	gactagtctattgcggcagggcgtcg	*S. meliloti*
LdtR_Smc__AgeI_Fw^c^	cgaccggtccctggcagtcgtctatcc	*S. meliloti*
LdtR_Smc__SpeI_Rv^c^	gactagtacaacctgcggcttcatct	*S. meliloti*
ΔLdtP_Smc_-1_Seq_Fw	atgtcgaaaaagaacggaa	*S. meliloti*
ΔLdtP_Smc_-2_Seq_Fw	agctcgagacaaacctggtg	*S. meliloti*
ΔLdtR_Smc__Seq_Fw	ggttcgcattgagctattgg	*S. meliloti*
Gus_seq_Rv	ccagacgttgcccgcataattacgaa	mini Tn5 Transposon
**pBBR1MCS-5 cloning**		
LdtR_Las__EcoRI_Fw^c^	ccggaattcgatgaacaataatatacaatcaaagataatttcg	‘*Ca.* L. asiaticus’
LdtR_Las__BamHI_Rv^c^	cgggatcctcaaagtcgatacgcaatctgatcaccc	‘*Ca.* L. asiaticus’
LdtP_Las__KpnI_Fw^c^	cggggtacccatggttggatatttaaagataaataaga	‘*Ca.* L. asiaticus’
LdtP_Las__EcoRI_Rv^c^	cggaattccgtcagtcagaatctataggatgatcctc	‘*Ca.* L. asiaticus’
**Quantitative RT-PCR**		
LdtR_Las__RTPCR_Fw	atttcggacacggatagtgc	‘*Ca.* L. asiaticus’
LdtR_Las__RTPCR_Rv	tgtaatcgctcaaccaaacg	‘*Ca.* L. asiaticus’
LdtP_Las__RTPCR_Fw	tcccgcggtctattattcag	‘*Ca.* L. asiaticus’
LdtP_Las__RTPCR_Rv	tccacttcctccacgaaaac	‘*Ca.* L. asiaticus’
CLIBASIA_00120(50S_L10)_RTPCR_Fw	tggaggtgtaaaagttgccaaa	‘*Ca.* L. asiaticus’
CLIBASIA_00120(50S_L10)_RTPCR_Rv	ccaacgaaaagatcagatattcctcta	‘*Ca.* L. asiaticus’
CLIBASIA_r05781(16S)_RTPCR_Fw	tcgagcgcgtatgcgaatacg	‘*Ca.* L. asiaticus’
CLIBASIA_r05781(16S)_RTPCR_Rv	gcgttatcccgtagaaaaaggtag	‘*Ca.* L. asiaticus’
LdtR_Las__ext_Rv	gcttgcacagcattcacatc	‘*Ca.* L. asiaticus’
LdtP_Las__ext_Rv	gaggctcaggactgttccaa	‘*Ca.* L. asiaticus’
CLIBASIA_00120(50S_L10)_ext_Rv	aaaaagatgcgggaagctg	‘*Ca.* L. asiaticus’
CLIBASIA_r05781(16S)_ext_Rv	accaaccagctaatccaacg	‘*Ca.* L. asiaticus’
LdtR_Lcr__RTPCR_Fw	aacgtcaaggccgtagtgat	*L. crescens*
LdtR_Lcr__RTPCR_Rv	cgagcgctgatgattaacaa	*L. crescens*
LdtP_Lcr__RTPCR_Fw	ggtggccacaggtggtaata	*L. crescens*
LdtP_Lcr__RTPCR_Rv	taacccatgtcgtgcttgaa	*L. crescens*
B488_08460_RTPCR_L10_Fw	tggctggtggttgtgttaag	*L. crescens*
B488_08460_RTPCR_L10_Rv	tttaggagcagcgactggat	*L. crescens*
LdtP_Lcr__ext_Rv	ccctgataccaaagcctgaa	*L. crescens*
LdtR_Lcr__ext_Rv	tctcctgcccaggcttagta	*L. crescens*
B488_08460_ext_Rv	cctttgcgaatctaacagca	*L. crescens*
16SLcr_Fw	gttcggaataactgggcgta	*L. crescens*
16SLcr_Rv	aaggttgagccttgggattt	*L. crescens*
16SLcr_ext_Rv	gcacctcagcgtcagtatca	*L. crescens*
Cox2_Fw	gtatgccacgtcgcattccaga	Universal Plant
Cox2_Rv	gccaaaactgctaagggcattc	Universal Plant
**Sequencing**		
M13_Fw	gttgtaaaacgacggccagt	*E. coli*
M13_Rv	aggaaacagctatgaccatg	*E. coli*
T7	taatacgactcactataggg	*E. coli*
T7 term	gctagttattgctcagcgg	*E. coli*

a Italics show the extra bases added to the 5′ end for the ligation independent cloning using the BD-infusion CF Dry-Down PCR cloning kit (BD Biosciences).

b Biotin labeled.

c Underlines indicate the enzyme restriction sites.

The DNA binding sequence for LdtR_Las_ in the promoter region of *ldtP_Las_* was identified by DNase I footprinting. The protected site consists of 18 nucleotides (ATATTCCTTGTATTTTAA, *ldtP_1*) on the minus strand (Fig. 2C), upstream of the predicted −35 box. Immediately downstream from the protected site, a 15 nt DNase I-hypersensitivity region was identified, which may correspond to a DNA bending site (Fig. 2C). Analysis of the DNA sequence upstream of the hypersensitivity region indicated the presence of a second binding site; however, the binding sequence is broken into two segments separated by 9 nt (ATATTTCTT-*n9*-GTGATTTAA, *ldtP_2*; Fig. 2D). A putative binding site was identified in the promoter region of *ldtR_Las_* with a similar disruption (*ldtR_1*, Fig. S2E). This sequence displays a separation of 6 nt between each segment, which may explain the lower affinity of LdtR_Las_ for *P_ldtR_* (Fig. 2A).

**Figure 3:**
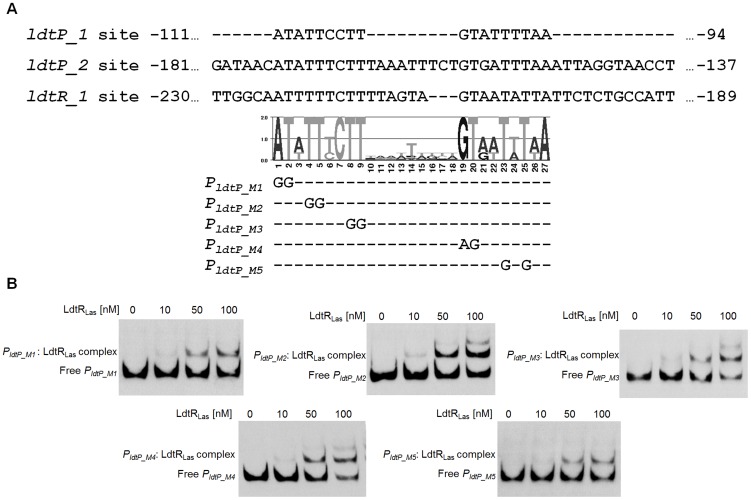
Characterization of LdtR_Las_ binding site. (A) Alignment of LdtR_Las_ binding sites *ldtP_1*, *ldtP_2*, and *ldtR_1*. Graphical representation (LOGO) of the position specific frequency matrix constructed with LdtR_Las_ binding sites. Double substitutions on the most conserved nucleotides were carried out in *P_LdtP_* probe. The location of each mutated nucleotide is depicted on probes *P_ldtP_M1_*, *P_ldtP_M2_*, *P_ldtP_M3_*, *P_ldtP_M4_*, and *P_ldtP_M5_*. (B) EMSAs were conducted on *P_ldtP_M1_*, *P_ldtP_M2_*, *P_ldtP_M3_*, *P_ldtP_M4_*, or *P_ldtP_M5_* probes with increasing concentrations of LdtR_Las_, as indicated on top of each panel. No protein was added to the first lane.

To determine the residues required for LdtR binding, the three binding sites (*ldtP_1*, *ldtP*_2, and *ldtR*_1) were compared, and a position specific frequency matrix was constructed (Fig. 3A). Using plasmid pBS6 as a template, site directed mutagenesis was performed on residues that showed high conservation as follow: M1 (−111)AT→GG, M2 (−108)TT→GG, M3 (−104)TT→GG, M4 (−102)GT→AG, and M5 (−99)TtT→GtG. EMSA experiments were then conducted with the mutated binding sites. Decreased LdtR_Las_ binding was observed with probes *P_ldtP_M1_* and *P_ldtP_M5_* (Fig. 3B). Mutations M2, M3, and M4 did not affect LdtR_Las_ binding (Fig. 3B).

### LdtR_Las_ is a transcriptional activator

**Figure 4:**
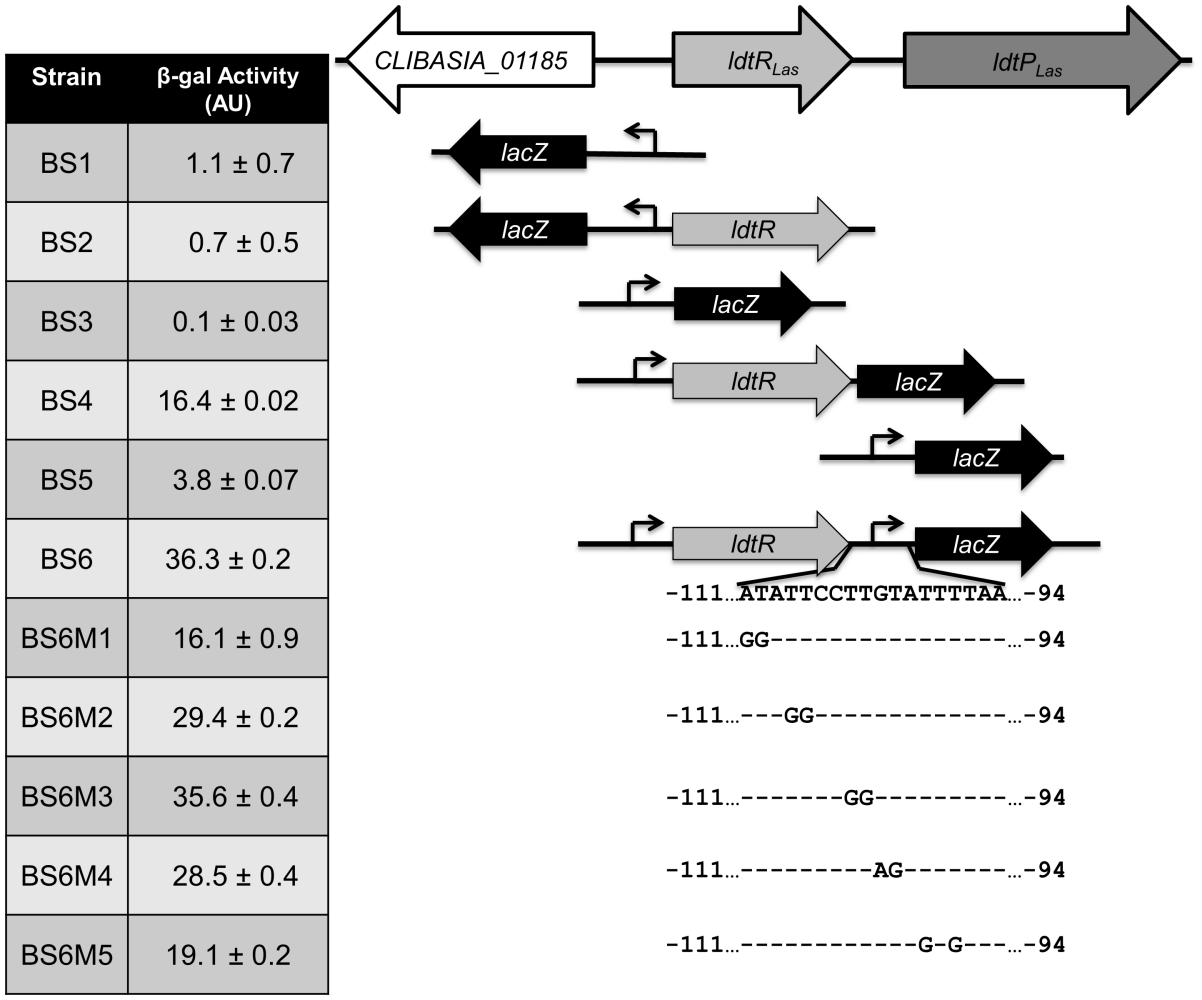
LdtR_Las_ is a transcriptional activator. Different transcriptional fusions to *lacZ* were constructed to evaluate *ldtR_Las_* and *ldtP_Las_* promoter activity in presence or absence of the transcriptional regulator, LdtR_Las_. β-galactosidase activity was determined at mid-exponential phase and expressed as arbitrary units (AU).

To determine the mode of regulation for LdtR_Las_, we generated several *lacZ* fusions using *Bacillus subtilis* as a model strain, since all the genes under study are absent from its genome. This system allows the study of transcriptional fusions by inserting a single copy of the gene into a non-essential chromosomal locus (*thrC*). The putative promoter regions of *CLIBASIA_01185*, *ldtR_Las_*, and *ldtP_Las_* were fused to the *lacZ* gene, resulting in strains BS1 (*P_CLIBASIA_01185_*), BS3 (*P_ldtR_*) and BS5 (*P_ldtP_*). All three promoters were found to have very low activity (1.1±0.7, 0.1±0.03 and 3.8±0.07 AU, respectively; Fig. 4). In the presence of *ldtR_Las_*, increased expression of *lacZ* was observed in strains BS4 (*P_ldtR_*-*ldtR_Las_*) and BS6 (*P_ldtR_*-*ldtR_Las_*-*P_ldtP_*) (16.4±0.02 and 36.3±0.2 AU, respectively; Fig. 4). No expression was observed in strain BS2 (harboring *ldtR_Las_* and *P_CLIBASIA_01185_*). These results confirmed that LdtR_Las_ is a transcriptional activator of *ldtR_Las_* and *ldtP_Las_*, while it does not regulate *CLIBASIA_01185*.

The *in vivo* specificity of LdtR_Las_ binding to *P_ldtR_*-*ldtR_Las_*-*P_ldtP_* was tested in strains BS6M1 and BS6M5, harboring mutations M1 or M5 on the *P_ldtP_* binding site 1. β-galactosidase activity was significantly reduced (p<0.001) by 55% and 47%, for BS6M1 and BS6M5 respectively, when compared to the wild type promoter (Fig. 4). These results positively correlated with the reduced binding of LdtR_Las_ to probes *P_ldtP_M1_* and *P_ldtP_M5_* in EMSA experiments (Fig. 3B), confirming the specificity of the LdtR_Las_ binding site.

### *ldtR-ldtP* mutations resulted in shortened cells and increased sensitivity to osmotic stress in *S. meliloti*

Inactivation of L,D-transpeptidases have been shown to induce morphological changes, resulting in decreased rigidity of the cell wall [8]. As ‘*Ca.* L. asiaticus’ has yet to be cultured, a model strain was used to study the biological role of LdtR and LdtP. Due to its close phylogenetic relationship to ‘*Ca.* L. asiaticus’, and the availability of genetic tools, *S. meliloti* was chosen. Prior to *in viv*o experiments, SMc01768 (named LdtR_Smc_) was purified and confirmed to bind to its own promoter region, as well as to the promoter region of the *ldtP_Las_* homolog, *SMc01769* (named *LdtP_Smc_*; Fig. S3).

Insertional mutants of *ldtP_Smc_* and *ldtR_Smc_* were constructed in *S. meliloti* (strains SMP1 and SMP2, respectively; Table 2) by homologous insertion of pSMP1 and pSMP2 in *ldtP_Smc_* and *ldtR_Smc_*, respectively. In strain SMP1, *ldtP_Smc_* was disrupted at 498 nt from the ATG start codon. In strain SMP2, *ldtR_Smc_* was disrupted 29 nt from ATG start codon.

**Table 2:** Strains and plasmids used in this study

Name	Relevant genotype	Origin/reference
**Bacterial Strains**		
*E. coli* DH5α	φ80 d*lacZ*ΔM15Δ(*lacZYA-argF*)U169 *recA1 endA1 hsdR17* (rk^−^. mk^+^) *supE44 thi-1 gyrA relA1*.	Laboratory stock
*E. coli* BL21 (DE3)	*F– ompT gal dcm lon hsdSB*(*rB- mB-*) *λ*(*DE3* [*lacI lacUV5-T7 gene 1 ind1 sam7 nin5]*).	Agilent Technologies
*B. subtilis* 168	*trpC2.*	*Bacillus* Genetic Stock Center
*S. meliloti* 1021	*expR102*::IS*Rm*2011-1 *expR*. Sm^r^.	[41]
*L. crescens* BT-1	Standard strain (Wild Type).	[13]
BS1	*B. subtilis* 168 *ΔthrC*:: [*PCLIBASIA_01185* (−395 to +47)*-lacZ*]. Em^r^.^a^	This work
BS2	*B. subtilis* 168 *ΔthrC*:: [*PCLIBASIA_01185-ldtR_Las_* (−395 to +516)*-lacZ*]. Em^r^.^a^	This work
BS3	*B. subtilis* 168 *ΔthrC*:: [*PldtR_Las_* (−395 to +47)*-lacZ*]. Em^r^.^a^	This work
BS4	*B. subtilis* 168 *ΔthrC*:: [*PldtR_Las_-ldtR_Las_* (−395 to +516)*-lacZ*]. Em^r^.^a^	This work
BS5	*B. subtilis* 168 *ΔthrC*:: [*PldtP_Las_* (+467 to +792)*-lacZ*]. Em^r^.^a^	This work
BS6	*B. subtilis* 168 *ΔthrC*:: [*PldtR_Las_-ldtR_Las_-PldtP_Las_* (−395 to +792)*-lacZ*]. Em^r^.^a^	This work
BS6M1	BS6 [−111 AT→GG]. Em^r^.^b^	This work
BS6M2	BS6 [−108 TT→GG ]. Em^r^.^b^	This work
BS6M3	BS6 [−104 TT→GG ]. Em^r^.^b^	This work
BS6M4	BS6 [−102 GT→AG ]. Em^r^.^b^	This work
BS6M5	BS6 [−99 TtT→GtG ]. Em^r^.^b^	This work
SMP1	*S. meliloti* 1021 *ldtP_Smc_* (+498)::*uidA*. Sm^r^. Neo^r^.	This work
SMP2	*S. meliloti* 1021 *ldtR_Smc_* (+29)::*uidA*. Sm^r^. Neo^r^.	This work
SMP3	*S. meliloti* 1021 *ldtP_Smc_* (+1332)::*uidA*. Sm^r^. Neo^r^.	This work
SMP2A	*S. meliloti* SMP2 pBBR1MCS-5. Sm^r^. Neo^r^. Gm^r^.	This work
SMP2B	*S. meliloti* SMP2 pSMP4. Sm^r^. Neo^r^. Gm^r^.	This work
SMP2C	*S. meliloti* SMP2 pSMP5. Sm^r^. Neo^r^. Gm^r^.	This work
**Plasmids**		
p15TV-L	Expression vector for purification of proteins by nickel affinity cromatography. Ap^r^.	Structural Genomics Consortium, University of Toronto
pDG1663	*B. subtilis* vector for ectopic integration into *thrC* site containing *E. coli spoVG-lacZ*. Ap^r^, Em^r^.	[31]
pRK600	Helper plasmid for triparental mating. pRK2013 Nm::Tn9. Cm^r^.	[35]
pVMG	pVO155 with stop codons upstream of *uidA* in all ORFs. Neo^r^.	[42]
pBS1	*P_CLIBASIA_01185_-lacZ* transcriptional fusion carrying ‘*Ca.* L. asiaticus’ *s*equence from −395 to +47 in pDG1663. Ap^r^, Em^r^.^a^	This work
pBS2	*P_CLIBASIA_01185_-ldtR-lacZ* transcriptional fusion carrying ‘*Ca.* L. asiaticus’ *s*equence from −395 to +516 in pDG1663. Ap^r^, Em^r^.^a^	This work
pBS3	*P_ldtR_-lacZ* transcriptional fusion carrying ‘*Ca.* L. asiaticus’ *s*equence from −395 to +47 in pDG1663. Ap^r^, Em^r^.^a^	This work
pBS4	*P_ldtR_-ldtR-lacZ* transcriptional fusion carrying ‘*Ca.* L. asiaticus’ *s*equence from −395 to +516 in pDG1663. Ap^r^, Em^r^.^a^	This work
pBS5	*P_ldtP_-lacZ* transcriptional fusion carrying ‘*Ca.* L. asiaticus’ *s*equence from +467 to +792 in pDG1663. Ap^r^, Em^r^.^a^	This work
pBS6	*P_ldtR_-ldtR-P_ldtP_-lacZ* transcriptional fusion carrying ‘*Ca.* L. asiaticus’ *s*equence from −395 to +792 in pDG1663. Ap^r^, Em^r^.^a^	This work
pBS6M1	pBS6 (−111) AT→GG. Ap^r^, Em^r^.^b^	This work
pBS6M2	pBS6 (−108) TT→GG. Ap^r^, Em^r^.^b^	This work
pBS6M3	pBS6 (−104) TT→GG. Ap^r^, Em^r^.^b^	This work
pBS6M4	pBS6 (−102) GT→AG. Ap^r^, Em^r^.^b^	This work
pBS6M5	pBS6 (−99) TtT→GtG. Ap^r^, Em^r^.^b^	This work
pSMP1	Internal 394 bp of *ldtP_Smc_* (from +104 to +498) in pVMG. Neo^r^.^b^	This work
pSMP2	407 bp of *PldtR_Smc_ and ldtR_Smc_* (from −378 to +29) in pVMG. Neo^r^.^a^	This work
pSMP3	*ldtP_Smc_-gus* transcriptional fusion in pVMG carrying *S. meliloti* sequence from +932 to +1332. Neo^r^.^b^	This work
pBBR1MCS-5	Broad host range vector. Gm^r^.	[9]
pSMP4	*ldtR*_Las_ (from +1 to +516) cloned into pBBR1MCS-5 (EcoRI/BamHI). Gm^r^.^a^	This work
pSMP5	*ldtP*_Las_ (from +1 to +1296) cloned into pBBR1MCS-5 (KpnI/EcoRI). Gm^r^.^b^	This work

a The positions indicated are relative to *ldtR* translational start site.

b The positions indicated are relative to *ldtP* translational start site.

**Figure 5:**
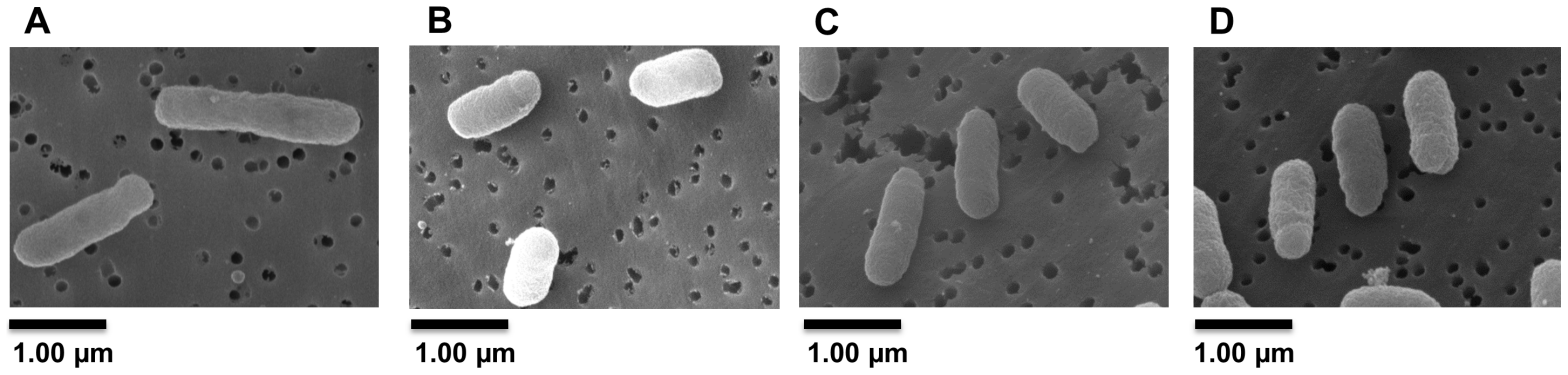
Inactivation of *ldtR_Smc_* or *ldtP_Smc_* results in a short-cell phenotype. Representative Scanning Electron Micrographs (SEM) of (A) wild type, (B) SMP1, and (C) SMP2 strains of *S. meliloti*. Cultures were grown in LB medium to mid-exponential phase and processed for SEM analysis to assess the morphological effects of *ldtR_Smc_* or *ldtP_Smc_* gene disruption. (D) Wild type strain grown in presence of 25 µM phloretin. Scale bar, 1.00 µm.

Analysis of crystal violet-stained cells revealed the SMP1 and SMP2 mutants had a shortened rod-type phenotype (short-cell), when compared to the wild type *S. meliloti*. However, they did not show growth defects in liquid cultures (doubling time or final OD_600_, data not shown). Scanning electron microscopy was used to verify and quantify these morphological changes. Electron micrographs confirmed the average length of SMP1 (1.16 µm±0.15) and SMP2 (1.15 µm±0.14) mutants to be significantly shorter (30%, p<0.005) than wild type cells (1.65 µm±0.20) (Fig. 5A–C).

**Figure 6:**
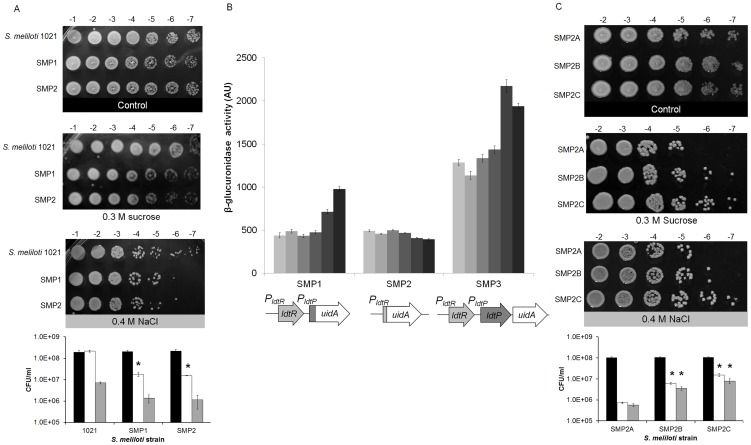
Inactivation of *ldtR_Smc_* or *ldtP_Smc_* increases sensitivity to osmotic stress. (A) Growth of wild type, SMP1, and SMP2 strains of *S. meliloti* on osmotic stress plates. Cultures were grown on LB medium until reached OD_600_ = 1.0, and then spot plated in serial dilutions, as indicated at the top of each panel. Pictures are representative of three biological replicates and were taken after 72 h of growth. Black bars correspond to control conditions, white bars 0.3 M sucrose, and grey bars 0.4 M NaCl. (* p<0.05). (B) The transcriptional activity of the different promoters was followed using the β-glucuronidase reporter in SMP1 and SMP2 disruption mutants (Table 2), as well as SMP3 strain (wild type-phenotype). Cultures were grown until mid-exponential phase in M9-glucose with increasing concentration of NaCl (9, 15, 50, 100, 170, and 250 mM; light gray to dark gray). β-glucuronidase activity was expressed as µM *p*-nitrophenol min^−1^ OD_600_^−1^. (C) Growth of SMP2A, SMP2B, and SMP2C strains of *S. meliloti* (Table 2) on osmotic stress plates. Cultures were grown on LB medium until reached OD_600_ = 1.0, and then spot plated in serial dilutions, as indicated at the top of each panel. Pictures are representative of three biological replicates and were taken after 72 h of growth. Black bars correspond to control conditions, white bars 0.3 M sucrose, and grey bars 0.4 M NaCl. (* p<0.05).

To determine if modifications in the cell wall composition would affect tolerance to osmotic stress, a seven-fold serial dilution of each strain was spot plated in the presence of sucrose (0.3 M) or NaCl (0.4 M). Increased sensitivity to osmotic stress was observed in strain SMP1 (1.8×10^7^±3.9×10^6^ and 1.4×10^6^±5.4×10^5^ CFU/ml, for sucrose and NaCl respectively), and SMP2 (1.6×10^7^±7.1×10^5^ and 1.1×10^6^±7×10^5^ CFU/ml, for sucrose and NaCl respectively), when compared to the wild type strain (2.2×10^8^±2.5×10^7^ and 7.1×10^6^±6.9×10^5^ CFU/ml, for sucrose and NaCl respectively; Fig. 6A). These results were significantly different for sucrose (p<0.05) but not for NaCl. Higher concentrations of NaCl or sucrose were toxic for all strains (data not shown).

To establish a link between elevated sensitivity to osmotic stress, and the regulation of gene expression by LdtR_Smc_, β-glucuronidase activity (encoded by the *uidA* gene) was measured in *S. meliloti* (Fig. 6B). Strain SMP3 was constructed by inserting the *uidA* reporter gene downstream of *ldtP_Smc_* (no disruption to *ldtR_Smc_* or *ldtP_Smc_*). Strain SMP3 was used as a reporter strain to determine the expression of *uidA* in a wild type phenotype. In the presence of NaCl, the β-glucuronidase activity was induced in a concentration-dependent manner in strains SMP3 and SMP1 (Fig. 6B). Induction of β-glucuronidase activity was dependent on the presence of LdtR_Smc_. In absence of the regulator (strain SMP2), no changes in the expression of the reporter gene were observed. These results confirm the role of LdtR_Smc_ as an activator of *ldtR_Smc_* and *ldtP_Smc_* transcription in response to osmotic stress.

To determine if the tolerance to osmotic stress could be recovered by the addition of *ldtR*, strain SMP2 (*ldtR* mutant) was transformed with plasmid pSMP4 carrying *ldtR_Las_* (strain SMP2B), and analyzed for sensitivity to osmotic stress. Strain SMP2A (carrying the empty pBBR1MCS-5 plasmid [9]) served as a control. Increased tolerance to osmotic stress was observed in strain SMP2B (6.1×10^6^±5.7×10^5^ and 3.6×10^6^±7.6×10^5^ CFU/ml, for sucrose and NaCl respectively p<0.05), when compared to SMP2A (7.2×10^5^±5.8×10^4^ and 5.7×10^5^±1.0×10^5^ CFU/ml, for sucrose and NaCl respectively) (Fig. 6C). These results suggest that LdtR_Las_ is directly involved in tolerance to osmotic stress by recognizing similar promoter elements in *P_ldtP_* of *S. meliloti*. Further *in silico* analyses in *S. meliloti* revealed the presence of LdtR_Las_ binding sites upstream of the *ldtP* −35 sequence, in agreement with the arrangement of LdtR binding sites in *L. crescens* and ‘*Ca.* L. asiaticus’ (Fig. S2).

To determine if the addition of *ldtP_Las_* could recover the tolerance to osmotic stress, strain SMP2 (*ldtR* mutant) was transformed with pSMP5 carrying *ldtP_Las_* (SMP2C). Increased tolerance to osmotic stress was observed in strain SMP2C (1.5×10^7^±2.6×10^6^ and 8.0×10^6^±2.1×10^6^ CFU/ml, for sucrose and NaCl respectively, p<0.05) when compared to SMP2A (7.2×10^5^±5.8×10^4^ and 5.7×10^5^±1.0×10^5^ CFU/ml, for sucrose and NaCl respectively, (Fig. 6C). These results indicate that LdtP from *S. meliloti* and ‘*Ca.* L. asiaticus’ are functionally homologous. Taken together these findings confirm that the decreased tolerance to osmotic stress observed in strain SMP2, was due to the absence of LdtR_Smc_ transcriptional activity.

### Identification of small molecules that modulate the activity of LdtR_Las_

A fluorescence based small molecule screening assay [10] was used to identify chemical scaffolds that may interact with the transcription factor LdtR_Las_. We utilized a library containing 196 biologically relevant small molecules [11], [12] and the Prestwick Chemical Library, which contains 1,200 small molecules [10]. Small molecules that induced a shift in the melting temperature (*ΔTm*) of LdtR_Las_, by more than two degrees, were considered as positive “hits”. The chemicals with the strongest destabilizing effect were hexestrol (*ΔTm* = −2.5±0.5°C), diethylstilbestrol (*ΔTm* = −4.5±0.9°C), and benzbromarone (*ΔTm* = −2.0±0.3°C), while oxantel pamoate (*ΔTm* = 2.0±0.2°C) was found to greatly increase the stability of the protein (Fig. S4).

### Small molecules decrease LdtR_Las_ binding to *P_ldtP_*

**Figure 7:**
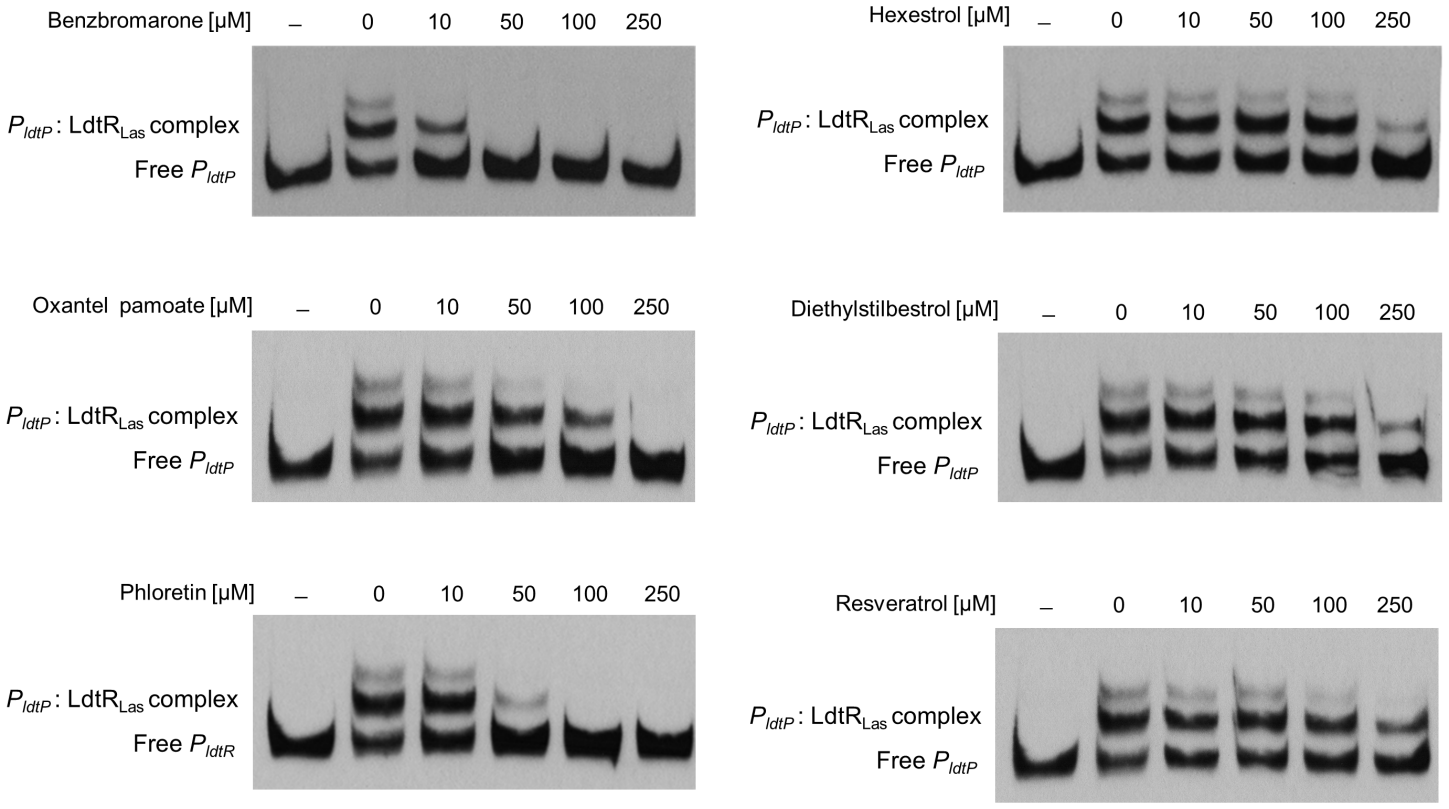
Small molecules decrease LdtR_Las_ binding to *P_ldtP_*. EMSAs were conducted in the presence of benzbromarone, hexestrol, oxantel pamoate, or diethylstilbestrol (identified in the screening assay), and phloretin or resveratrol, at the concentrations indicated on top of each panel. The concentration of LdtR_Las_ was maintained at 200 nM. No protein was added to the first lane.

A change in thermal stability does not guarantee a biologically relevant interaction; therefore, each of the compounds was tested on the ability to modulate the *P_ldtP_*: LdtR_Las_ interaction. All of the identified chemicals decreased the DNA binding activity of LdtR_Las_ in a concentration-dependent manner (Fig. 7). Benzbromarone had the strongest effect and disrupted the *P_ldtP_*: LdtR_Las_ interaction at 50 µM. Oxantel pamoate completely impaired the *P_ldtP_*: LdtR_Las_ interaction at 250 µM, where only partial disruption of the complex was observed with hexestrol and diethylstilbestrol. The chemical scaffold of the strongest destabilizing agents (benzbromarone and hexestrol) served to identify other natural compounds such as resveratrol and phloretin. It was found that resveratrol decreased binding at 250 µM, while phloretin disrupted the *P_ldtP_*: LdtR_Las_ interaction at 100 µM, consistent with molecules having physiological relevance (Fig. 7). To determine the specificity of each ligand that decreased *P_ldtP_*: LdtR_Las_ interaction, EMSA experiments were carried using a MarR homolog (LVIS0553), in the presence or absence of each chemical. The *P_LVIS0553_*: LVIS0553 interaction was previously found to be modulated by the presence of novobiocin [10]. As expected none of the identified ligands for LdtR_Las_ affected the binding of LVIS0553 to its cognate promoter (Fig. S5).

### Small molecules induce morphological changes in *S. meliloti* and *L. crescens*

**Figure 8:**
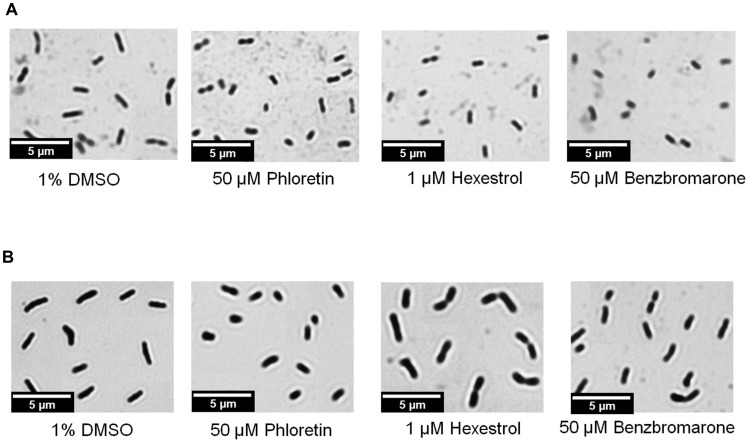
Short-cell phenotype as the result of *ldtR* inactivation by small molecules. Micrographs were taken after addition of benzbromarone, hexestrol, or phloretin to (A) *S. meliloti*, or (B) *L. crescens* cells. Samples were taken and stained with crystal violet. Magnification 600×, scale bar, 5.00 µm. Micrograph pictures are representative of three biological replicates.

We hypothesized that chemicals that modulate binding of the transcription factor would result in phenotypic abnormalities, similar to those observed in *ldtR* mutants of *S. meliloti*. The toxicity of each chemical was determined and sub-lethal concentrations were used for these experiments (Table 3). As expected, the addition of increasing concentrations of each chemical (25 µM phloretin, 25 µM benzbromarone, or 1 µM hexestrol) resulted in a pronounced decrease in cell size in *S. meliloti* (Fig. 8A). Quantitative assessments of the cell size were conducted in wild type *S. meliloti* cells grown in the presence of 25 µM phloretin. The addition of phloretin resulted in a significant decrease of 27% in the cell size (1.20 µm±0.18, p<0.005; Fig. 5D) when compared to the wild type (1.65 µm±0.20; Fig. 5A). These results are in agreement with the decrease in cell size observed for the SMP1 and SMP2 mutants (Fig. 5B and C).

**Table 3:** Minimal inhibitory concentrations (MICs) and small molecule concentration used for *in vivo* assays and plant experiments.

Chemical	Concentration tested^1^ (µM)			MIC (µM)	
	*L. crescens*	*S. meliloti*	Orange leaves	*S. meliloti*	*L. crescens*
Phloretin	25–100	25	100	50±4.2	150±9.1
Hexestrol	1–100	1	25	2±0.3	50±3.5
Benzbromarone^2^	10–50	25	100	50±5.3	>50

1 The concentration used for *in vivo* assays and plant experiments.

2 MIC for Benzbromarone was not determined due to poor solubility

Confirmatory studies were performed in *L. crescens* BT-1. *L. crescens* is a close relative of ‘*Ca.* L. asiaticus’ that was recently isolated from mountain papaya, and can be cultured under laboratory conditions. In addition, the complete genome of *L. crescens* BT-1 has been sequenced [13], and the homolog of *ldtR_Las_* (*B488_10910*, named *ldtR_Lcr_*) identified. The chemicals (50 µM phloretin, 50 µM benzbromarone, or 25 µM hexestrol) that induced the “short-cell” phenotype in *S. meliloti* modulated the activity of *ldtR_Lcr_*, resulting in a similar phenotype in *L. crescens* BT-1 (Fig. 8B).

To test if the phenotype induced by the presence of the chemicals correlated with changes in the expression of the *ldtR_Lcr_* and *B488_10900* (named *ldtP_Lcr_*), the mRNA levels were determined. *L. crescens* was grown to exponential phase, in presence or absence of the small molecules. Modest, but highly reproducible decreases of 45.4±8.9, 62.5±7.7, and 37.5±11.5 percent in *ldtP_Lcr_* expression, were observed upon growth in the presence of 25 µM phloretin, 50 µM benzbromarone, or 25 µM hexestrol, respectively. These results confirmed the role of the small molecules in modulating the activity of LdtR_Lcr_, *in vivo*.

### Small molecules decreased stress tolerance in *S. meliloti* and *L. crescens*

Based on the pivotal role of peptidoglycan in counteracting the effects of osmotic pressure, we hypothesized that the downregulation of *ldtR* and *ldtP*, by chemicals that impair LdtR activity, will result in decreased tolerance to osmotic stress.

**Figure 9:**
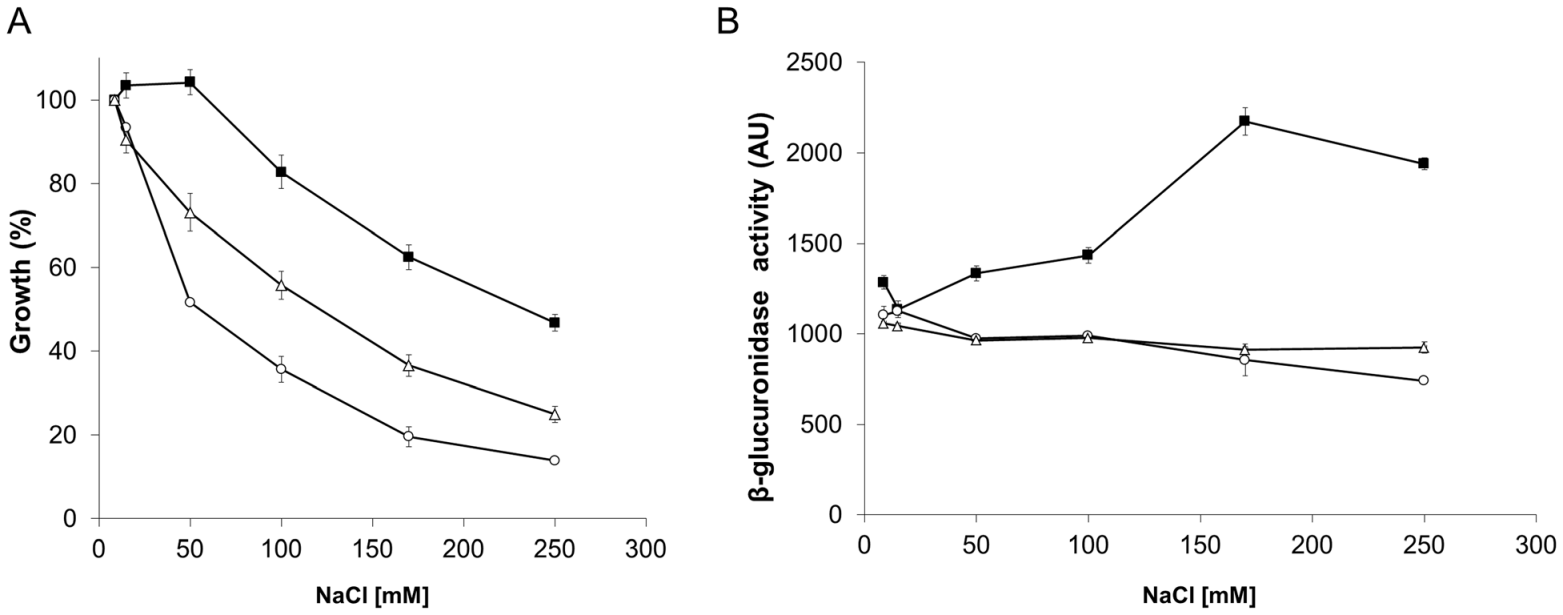
The inactivation of LdtR_Smc_ by small molecules reduces osmotic stress tolerance in *S. meliloti*. (A) SMP3 strain was grown in M9-glucose minimal medium with increasing concentrations of NaCl in absence (closed squares), or in presence of 25 µM benzbromarone (open triangles), or 25 µM phloretin (open circles). Growth was expressed as a percentage of the OD_600_ of cells grown in M9 media, at early stationary phase. The growth curves were performed in triplicates. (B) The induction the β-glucuronidase activity, as a response to osmotic stress (closed squares), was reduced in the presence of 25 µM benzbromarone (open triangles) or 25 µM phloretin (open circles). β-glucuronidase activity was expressed as µM *p*-nitrophenol min^−1^ OD_600_^−1^.

The *S. meliloti* wild type-phenotype strain, SMP3, was used to evaluate the effect of the small molecules, on the ability to grow under osmotic stress conditions. The cells were grown in the presence or absence of phloretin or benzbromarone with increasing concentrations of NaCl (Table 3; Fig. 9A). In the presence of the small molecules, strain SMP3 showed a severe decrease in tolerance to NaCl. At NaCl concentrations as low as 50 mM, a decrease in growth was observed in the presence of phloretin or benzbromarone (50 and 30%, respectively). Under these conditions, β-glucuronidase activity was determined. Induction of *ldtR_Smc_* and *ldtP_Smc_* expression, in response to high concentrations of NaCl, was overturned in presence of phloretin or benzbromarone (Fig. 9B). These results are in agreement with the decrease tolerance to osmotic stress observed in presence of the small molecules.

**Figure 10:**
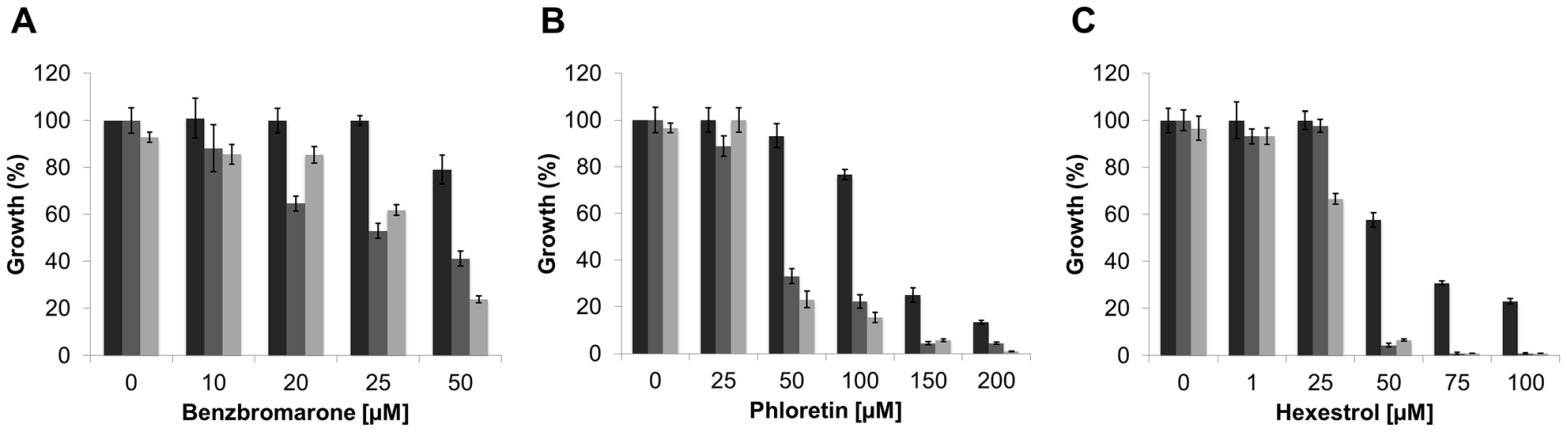
The inactivation of LdtR_Lcr_ by small molecules reduces osmotic stress tolerance in *L. crescens*. Cells were grown in modified BM7 media (black bars), supplemented with 200 mM sucrose (dark grey bars), or 150 mM NaCl (light gray bars). Increasing concentrations of (A) benzbromarone, (B) phloretin, or (C) hexestrol in the culture media are indicated under each panel. Growth was expressed as a percentage of the OD_600_ of cells grown in modified BM7 media, at stationary phase. The growth curves were performed in triplicates.

Since genetic tools are not available yet to manipulate *L. crescens*, we determined the effect of the addition of chemicals, at sublethal concentrations, on the ability to tolerate high concentrations of NaCl or sucrose. It was establish that the maximal concentration of NaCl and sucrose that *L. crescens* tolerate is 150 and 200 mM, respectively (Fig. S6). The effect of increasing concentrations of the small molecules was tested on the ability to tolerate NaCl or sucrose. The addition of phloretin, benzbromarone, or hexestrol (50, 100, or 25 µM, respectively), did not affect the growth of *L. crescens* in control conditions. Conversely, in the presence of NaCl or sucrose, *L. crescens* displayed increased sensitivity to all chemicals tested (Fig. 10). Together, these results indicate that in *S. meliloti* and *L. crescens*, tolerance to osmotic stress is in part mediated by changes in the peptidoglycan crosslinking, which can be manipulated by the addition of small molecules that modulate mRNA levels through LdtR activity.

### Small molecules as therapeutics

Based on these results, we designed an *in vitro* model to test the effectiveness of these chemicals. Shoots were collected from a single HLB-symptomatic Valencia Orange (*C. sinensis*) tree, infected with ‘*Ca.* L. asiaticus’. Previous studies have reported greater numbers of viable ‘*Ca.* L. asiaticus’ cells in the sieve elements of young, asymptomatic leaves, collected from new flushes [14]. All leaves used for this study were collected from new flushes on highly symptomatic branches. Nine leaves were collected for each treatment and control group. Samples were then incubated for 6 or 24 h (with or without chemical).

**Figure 11:**
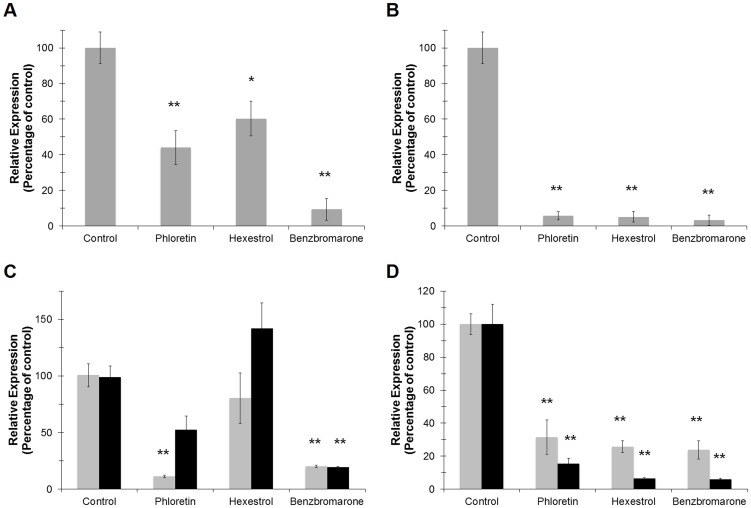
Small molecules modulate the transcriptional activity of LdtR_Las_ in infected sweet orange leaves. The expression levels of 16S RNA_Las_ (A) or *rplJ_Las_* (B) were assessed to monitor the viability of ‘*Ca.* L. asiaticus’ on the infected leaves upon treatment with the small molecules. The plant gene *cox2* was used to normalize the expression values between samples. The effect of the small molecules on the transcriptional activity of *ldtR_Las_* (C) or *ldtP_Las_* (D) genes was evaluated after 6 and 24 h (light and dark gray, respectively) of incubation with the small molecules. (* p<0.05; ** p<0.005).

Since ‘*Ca.* L. asiaticus’ still remains elusive to culture under laboratory conditions, we followed the transcriptional activity of the 16S RNA gene and the L10 ribosomal protein (encoded by the *rplJ* gene) as viability parameters. The amplification values were normalized to the plant gene *cox2* and are expressed relative to the control (incubated without chemical) samples. After 24 h of incubation, significant differences were observed in samples treated with small molecules. Expression of the 16S RNA gene was repressed in samples treated with hexestrol and phloretin [39.7±9.8 (p<0.05) and 55.9±9.5 (p<0.005) percent decrease, respectively], while benzbromarone showed the strongest effect, with 90.9±6.1 percent decreased expression (p<0.005) (Fig. 11A). A similar trend was observed for the expression of *rplJ*, with a decreased expression of 94.2±2.3, 94.6±2.9, and 97.6±1.5 percent for phloretin, hexestrol, and benzbromarone, respectively (p<0.005) (Fig. 11B). After a short period of incubation (6 h) no significant changes were observed (data not shown).

The effect of the chemicals on the expression of the specific genes *ldtR_Las_* and *ldtP_Las_* was then determined in the infected leaves. The expression values are calculated relative to the 16S RNA gene, to assess the specificity of the chemicals to target genes. Phloretin showed a strong effect (88.1±1.2 percent decrease) on the expression of *ldtR_Las_* after 6 h of incubation, while benzbromarone displayed similar decreased expression values after 6 or 24 h (78.9±1.3 and 80.5±1.1 percent, respectively, p<0.005; Fig. 11C). The expression of *ldtP_Las_* showed constant and incremental repression values over time. Hexestrol and benzbromarone reached maximal values of 93.7±0.8 and 94.2±0.9, respectively, while phloretin showed a maximal value of 84.8±3.8 percent decrease (p<0.005) (Fig. 11D).

These results indicate that the small molecules tested act specifically on the *ldtR_Las_* activator. We hypothesize that in ‘*Ca.* L asiaticus’, expression of LdtP is increased in response to osmotic stress, allowing persistence of the bacteria within the phloem of the tree. As such, the regulation of *ldtP* expression through inactivation of LdtR with small molecules represents a direct means of influencing osmotic stress tolerance, and survival of ‘*Ca.* L asiaticus’ within the host.

## Discussion

‘*Ca.* L. asiaticus’ is frequently exposed to changes in osmotic pressure, due to variations in phloem sap composition. Sucrose concentrations in the phloem can vary significantly (between 0.5 and 30% w/v, corresponding to 15 mM and 880 mM, respectively) depending on plant species, tissue, time of day, and season [15], [16]. Consequently, bacterial pathogens that replicate in the phloem must continuously respond to changes in osmotic pressure. In this context, L,D transpeptidase activity is critical, as these enzymes are directly involved in cell wall biosynthesis and remodeling in response to stress conditions.

In this report, we identified and characterized a regulon from the citrus pathogen ‘*Ca.* L. asiaticus’, involved in peptidoglycan remodeling. These results represent the first regulatory system functionally analyzed for this pathogen. Included in this regulon is *ldtR*, a member of the MarR family of transcriptional regulators, and *ldtP*, a predicted L,D-transpeptidase. The genomic context of *ldtR*_Las_ was conserved among members of the *Rhizobiacea* family. As such, the two closest phylogenic relatives of ‘*Ca.* L. asiaticus’, *S. meliloti* and *L. crescens*, were used to study the phenotypic effects of L,D-transpeptidase inactivation, and the physiological conditions that contribute to the expression of the *ldtR* regulon, since ‘*Ca.* L. asiaticus’ is yet to be cultured. The highly conserved nature of *ldtR* suggests a similar mechanism of regulation among these members of the *Rhizobiacea* family; however, the response to ligands may vary due to the different lifestyle of each species.

L,D-transpeptidases (E.C. 2.3.2.12) mediate the substitution of 4→3 (D-Ala^4^ to mDAP^3^) crosslinks, generated by the penicillin binding protein D,D-transpeptidase, to 3→3 (mDAP^3^ to mDAP^3^) crosslinks. This pattern of L,D-transpeptidation represents 80% of the crosslinks observed in the cell walls of stationary phase *M. tuberculosis* cells [17]. Similar results were observed in other microorganisms, including *E. coli* and *V. cholerae* [8], [18]. These observations suggest that transpeptidation is an active process in stationary phase cells, which may be critical for adaptation and tolerance to environmental stress. In *M. tuberculosis*, increased cell wall transpeptidation was positively correlated with increased transcription of Ldt_M1_ during nutrient starvation [17], [19]. Interestingly, our results in *L. crescens* indicate that *ldtP_Lcr_* and *ldtR_Lcr_* are expressed throughout the growth phases, when cultured under laboratory conditions. However, a comparative analysis of the ‘*Ca.* L. asiaticus’ transcriptome revealed that *ldtR* expression was five times higher in samples obtained from infected trees, when compared to samples collected from infected psyllids (an alternate host and insect vector of ‘*Ca.* L. asiaticus’) [20]. These results suggest that in ‘*Ca.* L. asiaticus’, transcription of Ldt-associated genes may be triggered by the high osmotic pressure generated by the phloem sap. These data, in combination with previous reports of the large proportion of 3→3 crosslinks in the muropeptides of Rhizobiales, suggest that LdtP may be involved in both housekeeping activities and stress response.

To further explore the LdtR regulatory mechanism, *Bacillus subtilis* was used as a heterologous host. Interestingly, we found that LdtR acts as a transcriptional activator of the *ldtR* and *ldtP* genes. Although the majority of MarR proteins act as transcriptional repressors, several MarR transcriptional activators have been described. In *S. meliloti*, the MarR family member ExpG binds to the ExpADGE operon to activate expression of the galactoglucan biosynthesis genes [21]. Similarly, PntR and PenR, from *Streptomyces arenae* and *S. exfoliatus*, respectively, activate synthesis of the pentalenolactone antibiotic [22]. Interestingly, all of these regulators bind AT-rich sequences similar to the binding sequence identified for LdtR [21]–[23]. This high degree of conservation could represent a common feature among binding sequences for MarR members that act as transcriptional activators.

In *S. meliloti*, changes in cell morphology (short-cell phenotype) were induced by the mutagenesis of *ldtR* and *ldtP*. Similar changes in cell morphology have been described for *S. meliloti* and *Rhizobium* spp in response to the accumulation of compounds such as glycine, which decreases the extent of crosslinks [24]–[26]. A similar short-cell phenotype was also observed in *V. cholerae*, following the accumulation of D-amino acids in the media [18]. Analysis of the ‘*Ca.* L. asiaticus’ genome revealed no homologs of the transpeptidases involved in these activities, however, a glutamate and alanine racemase were identified. These enzymes contribute to fluctuations in the concentration of D-amino acids. The potential involvement of LdtR in the regulation of these genes may explain the phenotypic changes observed in *ldtR* mutants. The direct or indirect involvement of LdtR in the regulation of these racemases is currently under examination.

Based on the biological relevance of the *ldtR* regulon, we identified small molecules (phloretin, benzbromarone, and hexestrol) that decreased binding of LdtR to its cognate promoters, resulting in decreased expression of *ldtP* and *ldtR*. In *L. crescens*, decreased gene expression in presence of these small molecules was positively correlated with decreased tolerance to osmotic stress. Furthermore, in *S. meliloti*, the addition of phloretin, benzbromarone, or hexestrol resulted in morphological changes (short-cell phenotype) similar to those observed in *ldtR* and *ldtP* mutants. Consequently, we reasoned that chemical manipulation of LdtR_Las_ activity will reduce long term survival and persistence of the pathogen in infected citrus trees. Thus, we designed an *in vitro* model using sweet orange leaves infected with ‘*Ca.* L. asiaticus’, to validate the effect of these chemicals. In samples treated with the small molecules, a significant decrease in *ldtR* and *ldtP* expression was observed, confirming the specific effect of these chemicals in ‘*Ca.* L asiaticus’. The use of a specific target is essential for the development of an effective therapeutic treatment. Modulation of cell wall transpeptidation has been used as a therapeutic treatment for recalcitrant microorganisms, such as *Mycobacterium tuberculosis* [27]. In contrast, current efforts towards the treatment of Huanglongbing disease are focused primarily on the use of “broad spectrum” treatments (i.e. penicillin, streptomycin, and thermotherapy). This study provides strong proof of concept for the use of small molecules that target LdtR_Las_, as a potential treatment option for Huanglongbing disease.

## Materials and Methods

### Strains and growth conditions

Bacterial strains and plasmids are listed in Table 2. *Escherichia coli* and *Bacillus subtilis* strains were grown in Luria-Bertani (LB) medium at 37°C. *S. meliloti* cells were grown at 30°C in either LB medium or M9 minimal medium with glucose. When required, the media was supplemented with gentamicin (30 µg ml^−1^), ampicillin, (100 µg ml^−1^), or chloramphenicol (170 µg ml^−1^) for *E. coli*; neomycin (100 µg ml^−1^), gentamicin (30 µg ml^−1^), and streptomycin (250 µg ml^−1^) for *S. meliloti*; or with erythromycin (1 µg ml^−1^) for *B. subtilis*.

*L. crescens* BT-1 was cultured at 25°C, with moderate agitation (150 rpm), in modified BM7 media [13] containing 1% Brain Heart Infusion (Difco Laboratories, Detroit, MI), 15% Fetal Bovine Serum (Sigma-Aldrich, St. Louis, MO), 30% TMN-FH insect medium (Sigma), α-Ketoglutaric acid (2 mg ml^−1^), ACES (10 mg ml^−1^), and potassium hydroxide (3.75 mg ml^−1^), at pH 6.9. Sodium chloride (0–200 mM) or sucrose (0–300 mM) was added to the growth media to induce osmotic stress. All antibiotics and chemicals were purchased from Sigma-Aldrich.

### DNA manipulations

Standard methods were used for chromosomal DNA isolation, restriction enzyme digestion, agarose gel electrophoresis, ligation, and transformation [28]. Plasmids were isolated using QIAprep Spin Miniprep Kit (Qiagen, Valencia, CA), and PCR products were purified using Qiaquick purification kits (Qiagen).

For protein expression and purification, *ldtR* gene was amplified from ‘*Ca.* L. asiaticus’ str. psy62 or *S. meliloti* 1021 chromosomal DNA via PCR, and then cloned into the p15TV-L plasmid as described previously [10].

### Protein purification

Protein expression and purification was performed as previously described [10]. Concisely, the His-tagged fusion proteins were overexpressed in *E. coli* BL21-Star(DE3) cells (Agilent Technologies, Santa Clara, CA). The cells were grown in LB medium at 37°C to an OD_600_ = 0.6 and expression induced with 0.5 mM isopropyl β-D-1-thiogalactopyranoside (IPTG). After addition of IPTG, the cells were incubated with shaking at 15°C overnight. The cells were harvested and resuspended in binding buffer (500 mM NaCl, 5% glycerol, 50 mM Tris pH 8.0, 5 mM imidazole, 0.5 mM TCEP), and stored at −80°C. The thawed cells were lysed and passed through a French Press. The lysate was clarified by centrifugation (30 min at 17,000 rpm at 4°C) and applied to a metal chelate affinity-column charged with Ni^2+^. After the column was washed, the protein was eluted from the column using elution buffer (binding buffer with 250 mM Imidazole). The hexa-histidine tag was then cleaved from the protein by treatment with recombinant His-tagged TEV protease. The cleaved protein was then resolved from the cleaved His-tag and the His-tagged protease by passing the mixture through a second Ni^2+^-column. The purified proteins were dialyzed against 10 mM Tris pH 8.0, 500 mM NaCl, 0.5 mM TCEP, and 2.5% glycerol. Finally, the proteins were aliquoted and stored at −80°C.

### Small molecule screening by differential scanning fluorimetry

Purified LdtR protein was screened against a library of 160 intracellular compounds [11] at a final concentration of 100 µM, or against the Prestwick chemical library of 1152 compounds (Prestwick Chemical, France) at a final concentration of 1.3 µg/mL, using fluorometry as previously described [11], [29]. LdtR was diluted to a final concentration of 30 µM in 100 mM Tris pH 8.0, 150 mM NaCl. SYPRO orange was added to a final concentration of 5×. 25 µL aliquots of protein solution containing the chemical compounds were placed in duplicate into 96 well plates (Bio-Rad, Hercules, CA) and heated from 25°C to 80°C at the rate of 1°C per minute. A real-time PCR device (iCycler IQ, Bio-Rad) was used to monitor protein unfolding by the increase in the fluorescence of the fluorophor SYPRO Orange (Life Technologies, Grand Island, NY). Fluorescence intensities were plotted against temperature for each sample well and transition curves were fitted using the Boltzmann equation using Origin 8 software (Northampton, MA). The midpoint of each transition was calculated and compared to the midpoint calculated for the reference sample. If the difference between them was greater than 2.0°C, the corresponding compound was considered to be a “hit” and the experiment was repeated to confirm the effect in a dose dependent manner. Figure S4 shows the melting curves obtained for LdtR_Las_ without chemicals or in presence of the selected hit chemicals. The chemicals that were not selected displayed melting curves similar to the one observed for the control.

### Electrophoretic Mobility Shift Assays (EMSAs)

Gel shift assays for LdtR were performed using aliquots of protein purified and concentrated according to the procedures described above. Fragments of the *ldtR*, and *ldtP* promoters were generated by PCR using biotin prelabeled (5′-end) primers (Table 1), then purified using QIAquick spin columns (Qiagen). Incubation mixtures for EMSA (20 µL) contained 1 ng of a 5′-labelled DNA probe, 50 mM Tris-HCl pH 7.2, 150 mM KCl, 10 mM MgCl_2_, 0.01% Triton ×100, 12.5 ng/µL of both Poly(dI-dC) and Poly(dA-dT) nonspecific competitor DNAs, purified LdtR protein (0–400 nM), and ligand (0–1 mM) when indicated. After incubation for 20 min at 37°C, samples were separated on 6% acrylamide-bisacrylamide nondenaturing gels in 0.5× Tris borate-EDTA buffer, pH 8.3 (TBE). Electrophoresis was performed at 100 V using ice-cold 0.5× TBE as a running buffer. DNA was then transferred from the polyacrylamide gel to a Hybond-N^+^ membrane (GE Healthcare, Pittsburgh, PA) by electroblotting at 250 mA for 45 min in a semidry transfer. Transferred DNA was cross-linked for 15 min using a UV cross-linker equipped with 312 nm bulbs. Biotin labeled DNA was detected using a Phototope-Star Detection Kit (New England Biolabs, Ipswich, MA). Membranes were exposed to Kodak X-ray film.

For the EMSA competitions assays, different fragments of the promoter regions were synthesized using PCR, or by annealing of primers as previously described [10] (Table 1).

### DNase I footprinting

Protection assays were performed on both minus and plus strands using 5′-6FAM or 5′-VIC labeled probes generated by PCR using primers described in Table 1. The protection assay contained the same components used for EMSAs, except that 5 ng µl^−1^ *P_ldtP_* labeled probe, 6 µM LdtR_Las_, 0.5 mM CaCl_2_, 2.5 mM MgCl_2_, and 0.025 U of DNase I (New England Biolabs) were added into 200 µL of reaction. The mix was incubated for 20 min at 37°C, and ended by adding 50 mM EDTA pH 8.0. The corresponding digestion reaction without LdtR was included as a control. The digested DNA and the sequencing reaction products were analyzed at the Plant and Microbe Genomics facility, Ohio State University, Columbus, using a 3730 DNA analyzer. The protected regions were identified using GeneMapper software (Life Technologies), as previously described [30].

### 5′RACE-PCR

The transcription start site of *ldtR* and *ldtP* genes from ‘*Ca.* L. asiaticus’ and *L. crescens* were determined by a modified 5′RACE-PCR protocol. Cultures of *B. subtilis* BS6 (for ‘*Ca.* L. asiaticus’ *ldtR* and *ldtP*) and *L. crescens* were grown to exponential phase as described above. The total RNA was extracted using the RiboPure-Bacteria kit (Ambion, Austin, TX) following the manufacturer's protocol. 2.5 µg of each RNA was first treated with 20 U of the Calf intestine alkaline phosphatase (New England Biolabs) for 1 h to remove the 5′-PO_4_ from degraded RNAs followed by a phenol∶chloroform∶isoamylalcohol precipitation. The RNAs were further treated with 2.5 U of Tobacco acid pyrophosphatase (Epicentre Biotechnologies, Madison, WI) for 1 h to remove the 5′-cap from mRNAs. The CIP/TAP RNAs were then ligated to the Oligo_RACE_RNA adapter (Table 1). The synthesis of the first strand of cDNAs were carried out using primers described in Table 1, with the SuperScript II Reverse Transcriptase (Invitrogen) and according to the manufacturer's protocol. The cDNAs were amplified by PCR using Oligo_RACE_Fw and LdtR_Las__RACE_Rv or LdtP_Las__RACE_Rv for ‘*Ca.* L. asiaticus’. Similarly, Oligo_RACE_Fw and LdtR_Lcr__RACE_Rv or LdtP_Lcr__RACE_Rv were used for *L. crescens* (Table 1). The PCR fragments were cloned using the StrataClone Blunt PCR cloning kit (Agilent Technologies), following the manufacturer's protocol. The clones were sequenced and *ldtR* and *ldtP* transcriptional start sites determined.

### Size-exclusion chromatography

100 µl of protein samples were prepared using 10 mM Tris pH 8.0, 500 mM NaCl, and 10 µM LdtR_Las_. The sample was incubated 20 min on ice and then injected onto a prepacked Superose 12 10/300 GL gel filtration column (GE Healthcare), connected to a LCC-501 plus (GE Healthcare), and equilibrated with 10 mM Tris pH 8.0 and 500 mM NaCl. Filtration was performed in a flow rate of 0.5 ml/min at 4°C. The eluted protein was monitored continuously for absorbance at 280 nm using a UV-M II monitor (GE Healthcare). Blue dextran 2000 was used to determine the void volume of the column. A combination of protein molecular weight standards, including IgG (150 kDa), BSA (66 kDa), Albumin (45 kDa), Trypsinogen (24 kDa), Cytochrome C (12.4 kDa), and Vitamin B12 (1.36 kDa) was also applied to the column under the same conditions. The elution volume and molecular mass of each protein standard was used to elaborate a standard curve for further determination of the molecular weight of the proteins under study. The theoretical molecular weight of LdtR was calculated from the amino acid sequence using the Compute pI/Mw tool at the ExPASy Proteomics Server (http://ca.expasy.org/tools/pi_tool.html).

### Construction of *lacZ* fusions and β-galactosidase assays

Plasmid pDG1663 [31] was used for the transcriptional analysis of *ldtR* expression. Plasmids pBS1, pBS2, pBS3, pBS4, pBS5, and pBS6 described in Table 2, were constructed using primers listed in Table 1. To this end, the PCR fragments were cut with HindIII and BamHI restriction enzymes, and ligated into pDG1663 previously digested with the same restriction enzymes. The recombinant clones selected in *E. coli* DH5α were confirmed by sequencing with primer pDGseq9_Fw. Plasmids pBS6M1, pBS6M2, pBS6M3, pBS6M4, and pBS6M5 were constructed by site-directed mutagenesis in pBS6 using the QuikChange Site-directed Mutagenesis kit (Agilent Technologies). The primers used are listed in Table 1. The transfer of plasmids pBS1, pBS2, pBS3, pBS4, pBS5, pBS6, pBS6M1, pBS6M2, pBS6M3, pBS6M4, and pBS6M5 into *B. subtilis* 168 was carried out by natural competence [32]. The new generated strains are listed and detailed in Table 2. The integration into the *thrC* locus was confirmed via extraction of *B. subtilis* genomic DNA using DNeasy Blood and Tissue kit (Qiagen), followed by PCR with primers pDGseq9_Fw and pDGseq10_Rv (Table 1).

For the β-galactosidase assays, *B. subtilis* cells were grown at 37°C in LB medium until reached an OD_600_ of 0.3 (mid-exponential phase). Cells were collected and washed twice with 0.9% NaCl, and permeabilized with 1% toluene in Z-buffer (60 mM Na_2_HPO_4_, 40 mM NaH_2_PO_4_, 10 mM KCl, 1 mM MgSO_4_, 50 mM β-mercaptoethanol) [33]. β-galactosidase activity was assayed by following the catalytic hydrolysis of chlorophenol red-β-D-galactopyranoside (Sigma-Aldrich). The absorbance at 570 nm was read continuously using a Synergy HT 96-well plate reader (BioTek, Winooski, VT). β-galactosidase activity, expressed as arbitrary units (AU), was calculated using the slope of absorbance curve normalized with the initial cell density. The assays were performed in triplicates.

### Construction of reporter, gene disruption and complemented strains of *S. meliloti* and β-glucuronidase assays

Promoter fusions to the *uidA* reporter gene, as well as *ldtR_Smc_* and *ldtP_Smc_* disruption mutants, were generated using plasmid pVMG [34]. pVMG is a modified version of plasmid pVO155, containing a multiple cloning site upstream of a promoterless β-glucuronidase (*uidA*) reporter gene [35]. For the generation of recombinant strains, a ∼400 bp region of the target gene (−378 to +29 of *ldtR_Smc_*, +104 to +498 of *ldtP_Smc_*, and +932 to +1332 of *ldtP_Smc_*, for pSMP2, pSMP1, and pSMP3, respectively) was amplified by PCR using the primers detailed in Table 1. The amplified fragments were inserted into the SpeI and AgeI restriction sites upstream of *uidA*, in pVMG. The resultant plasmids were propagated in DH5α and mobilized into *S. meliloti* 1021 via triparental mating, using helper plasmid pRK600 [36]. Transconjugants were selected on M9 sucrose-neomycin plates and their correct insertion confirmed by sequencing, using primers upstream of the original fragment used for cloning into pVMG and primer Gus_Seq_Rv, located 204 bp inside *uidA* reporter gene (Table 1).

For complementation assays, the complete sequence of *ldtR_Las_* gene was amplified by PCR using primers LdtR_Las__EcoRI_Fw and LdtR_Las__BamHI_Rv, while *ldtP_Las_* sequence was amplified using primers LdtP_Las__KpnI_Fw and LdtP_Las__EcoRI_Rv (Table 1). The DNA fragments were inserted into pBBR1MCS-5 plasmid, previously digested with the corresponding restriction enzymes, generating plasmids pSMP4 (*ldtR_Las_*) and pSMP5 (*ldtP_Las_*). The recombinant plasmids were selected in DH5α and confirmed by sequencing using universal M13 primers (Table 1). Plasmids pBBR1MCS-5, pSMP4, and pSMP5 were mobilized into *S. meliloti* SMP2 via triparental mating, using helper plasmid pRK600 [36]. Transconjugants were selected on M9 sucrose-neomycin-gentamicin plates.

For the β-glucuronidase assays, *S. meliloti* cells were grown in M9 minimal media, supplemented with NaCl, phloretin, or benzbromarone when indicated, until reached late-exponential phase. Cells were collected and washed twice with 0.9% NaCl, and permeabilized with 1% toluene in Z-buffer (60 mM Na_2_HPO_4_, 40 mM NaH_2_PO_4_, 10 mM KCl, 1 mM MgSO_4_, 50 mM β-mercaptoethanol) as previously described [33]. β-glucuronidase activity was measured by means of the hydrolysis of 4-nitrophenyl β-D-glucuronide substrate (Sigma-Aldrich). The absorbance at 405 nm was read continuously using a Synergy HT 96-well plate reader (BioTek). β-glucuronidase activity was expressed as µM of *p*-nitrophenol generated per min, normalized with the initial cell density. The assays were performed in triplicates.

### qRT-PCR studies

*L. crescens* cells were cultured in broth with hexestrol (25 µM), phloretin (50 µM), or benzbromarone (50 µM) when required. The cells were collected by centrifugation at 4°C when OD_600_ = 0.3 (mid-exponential phase). Total RNA was subsequently isolated with RiboPure-Bacteria (Ambion) in accordance with the manufacturer's protocol. cDNAs were synthesized with the Superscript first-strand synthesis kit (Life Technologies) in accordance with the manufacturer's instructions and stored at −80°C prior to use. Real-time quantitative PCR (qRT-PCR) was carried out in a iCycler IQ apparatus (Bio-Rad) using Platinum SYBR Green qPCR SuperMix for iCycler (Life Technologies) in accordance with the manufacturer's recommended protocol. Primers used for the qRT-PCR are described in further detail on Table 1. The RNA polymerase sigma factor *rpoD*, 50S ribosomal protein *L10*, 50S ribosomal protein *L12* genes, (*B488_13350*, *B488_08460*, *B488_08450*, respectively), *and 16S ribosomal RNA* were used as internal controls.

### Stress resistance assays

To test resistance to NaCl and sucrose, *S. meliloti* cells were grown in LB media to exponential phase (OD_600_ = 1.0). Serial dilutions were made and 4 µl was spot plated. Plates were prepared to contain 0.4 M NaCl or 0.3 M sucrose. In *L. crescens* the effect of chemical inactivation of LdtR on the stress tolerance was tested by following growth (as increased optical density) on liquid cultures.

### Scanning Electron Microscopy (SEM)

The morphology of different strains of *S. meliloti* (Table 2) was visualized by scanning electron microscopy using a Hitachi S-4000 FE-SEM apparatus (ICBR Electron Microscopy Core Lab, University of Florida, FL). *S. meliloti* 1021 strain, grown in the presence or absence of 25 µM phloretin, as well as SMP1 and SMP2 mutants, were cultured until exponential phase (OD_600_ = 1.0) in LB media, as described above. Prior fixation, the cells were centrifuged 3 min at 8,000 rpm and the pellets washed twice with 1× PBS buffer. Finally, the cells were treated with 1 mL of Trump's fixative solution for 20 min at room temperature, and post-fixed in 1% osmium tetroxide followed by dehydration in graded ethanol concentrations, following Electron Microscopy Core Lab recommended procedures. For the statistical analysis, the size of 10 cells per strain per field was determined (6 fields per strain).

### *In vitro* model to test chemicals on ‘*Ca.* L. asiaticus’ infected leaves

#### Source of leaves

All leaves used in this study were collected from young flushes that grew on highly symptomatic branches. All shoots were collected from a single HLB-infected, Valencia sweet orange (*C. sinensis*) tree, maintained by the University of Florida Citrus Research and Education Center (CREC) in Lake Alfred, FL. The infected status of the tree, and widespread distribution of ‘*Ca.* L. asiaticus’, were confirmed by transmission electron microscopy and PCR analysis of leaf, petiole, and root tissue samples, as described [14], [37]–[39].

#### Leaf collection and treatment

All solutions were autoclaved or filter sterilized. 100 mM stocks of each chemical were prepared in 100% DMSO. Immediately before collecting leaves, benzbromarone, hexestrol, and phloretin solutions were diluted to 100 µM in ultrapure water. A solution of ultrapure water and DMSO (1%) was used for the controls. A scalpel was used to harvest leaves from the tree, with a horizontal cut at the base of the petiole. Each leaf was immediately suspended in 8 ml of treatment solution (with or without chemicals). Leaves were supported in a vertical position throughout the incubation period, with only the lower inch of the petiole submerged in solution (with or without the chemical). Steady air flow was maintained over the leaf blades throughout the incubation period to facilitate transpiration and the uptake of each solution. Each treatment group consisted of 18 leaves. Nine leaves from each treatment group (including controls) were processed after 6 h of incubation, and the remaining nine leaves were processed after 24 h of incubation.

#### Leaf tissue processing

For each treatment group, biological triplicates (A, B and C) were prepared from nine leaves. Due to the variable distribution of ‘*Ca.* Liberibacter asiaticus’ within host trees [37], [40], the tissue from three leaves was combined for each sample. RNA extractions were carried out using the midribs and petioles only. The leaf blades were removed using a scalpel. The remaining midrib and petiole of each leaf was cut into sections (1 cm long) and immediately submerged in RNA*later* solution (Life Technologies) as per the manufacturer's instructions. The samples were stored at −80°C until being processed for RNA isolation.

#### RNA extraction

Plant and bacterial RNA was extracted from midrib and petiole samples using TRI Reagent solution (Sigma-Aldrich), with the addition of a mechanical homogenization step and pressure lysis. Midrib and petiole samples were thawed on ice, and transferred to FT500-S Pulse Tubes (Pressure Biosciences, Easton, MA) with 500 µl of TRI Reagent (Sigma-Aldrich). Samples were homogenized for a total of 2 minutes, in 30 s intervals, on ice, using a PCT Shredder (Pressure Biosciences, Easton, MA). Samples were then transferred to FT500-ND Pulse Tubes (Pressure Biosciences) and subjected to pressure cycling using a NEP 2320 Barocycler (Pressure Biosciences) at 35,000 psi for 30 s, and 0 psi for 30 s, for a total of 20 cycles. Crude lysate was then centrifuged at 5,000× g for 5 min, at 4°C, and the supernatant transferred to a clean RNase free falcon tube for RNA extraction. Chloroform (0.2 volumes) was added to each sample followed by thorough mixing and centrifugation at 5,000× g for 30 min, at 4°C. The aqueous phase was transferred to a clean RNase free falcon tube, and precipitated with isopropanol (0.5 volumes). RNA pellets were washed with 75% ethanol (0.75 vol), briefly air dried, and re-suspended in 100 µl of RNase-free water. RNA samples were treated with RNase-free DNase I for 30 min, at 37°C, followed by DNase Inactivation Reagent (Life Technologies). The concentration of total isolated RNA was determined using a NanoDrop ND 1000 (Thermo Scientific, Wilmington, DE). RNA samples were stored at −80°C. cDNAs were synthesized with M-MLV Reverse Transcriptase (Life Technologies) in accordance with the manufacturer's instructions, using the primers listed in Table 1. Real time quantitative PCR (qRT-PCR) was carried out on as described above. The *cox2* gene was measured as an internal plant control. Quantitative reverse transcription-PCR primers are described in detail in Table 1.

### Statistical analyses

The statistical significance of data obtained from SEM (cell size) and stress resistance assays (CFU/ml), was determined using a Student's *t*-test. qRT-PCR statistical significance was assessed using a two-tail P-value, calculated with the Mann–Whitney nonparametric test.

## Supporting Information

Figure S1 **Determination of the oligomeric state of LdtR_Las_.** Size-exclusion chromatography was performed using a Superose 12 column, as described in Materials and Methods section.
(TIF)

Figure S2 **The location of LdtR binding sites in** ***P_ldtR_*** **and** ***P_ldtP_*** **is conserved upstream of the promoter elements in ‘** ***Ca.*** **L. asiaticus’,** ***L. crescens*** **, and** ***S. meliloti*** **.** (A) Graphical representation (to scale) of the LdtR binding sites and promoter elements of *ldtP* in ‘*Ca.* L. asiaticus’, *L. crescens*, and *S. meliloti*. Detailed characterization of *P_ldtP_* in (B) *L. crescens*, or (C) *S. meliloti*. (D) Graphical representation (to scale) of the LdtR binding sites and promoter elements of *ldtR* in ‘*Ca.* L. asiaticus’, *L. crescens*, and *S. meliloti*. Detailed characterization of *P_ldtR_* in (E) ‘*Ca.* L. asiaticus’, (F) *L. crescens*, or (G) *S. meliloti*. The experimentally determined transcription start site (+1) of *ldtP* and *ldtR* in ‘*Ca.* L. asiaticus’ and *L. crescens*, as well as the predicted transcription start site in *S. meliloti* are depicted in a triangle. The −10 and −35 boxes, as well as the Shine-Dalgarno sequence (SD) are underlined and highlighted in gray boxes. The putative binding sites for LdtR are identified by dashed boxes.
(TIF)

Figure S3 **LdtR_Smc_ binds to** ***P_ldtR_*** **and** ***P_ldtP_*** **of** ***S. meliloti*** **.** EMSAs were conducted on (A) *P_ldtR_* or (B) *P_ldtP_* probes with increasing concentrations of LdtR_Smc_, as indicated on top of each panel. No protein was added to the first lane.
(TIF)

Figure S4 **Small molecules induce a shift in the thermal stability of LdtR_Las_.** The melting curves of purified LdtR_Las_ [30 µM] are depicted in absence (no marker) or presence of ligands: oxantel pamoate (diamonds), benzbromarone (triangles), diesthylstilbestrol (circles), and hexestrol (squares).
(TIF)

Figure S5 ***P_LVIS0553_:*** **LVIS0553 interaction is not affected by LdtR_Las_ ligands.** EMSAs were conducted in the presence of 200 µM benzbromarone, hexestrol, oxantel pamoate, diethylstilbestrol, phloretin or resveratrol. The concentration of LVIS0553 was maintained at 20 nM. No protein was added to the first lane.
(TIF)

Figure S6 **Growth of** ***L. crescen*** **s with increasing concentrations of NaCl or sucrose.** In A, sucrose was added at 0 (empty square), 100 (diamond), 200 (triangle), 400 (circle) or 600 (filled square) mM. In B, NaCl was added at 0 (empty square), 100 (diamond), 150 (triangle), 200 (circle) or 400 (filled square). The growth curves were performed in triplicates.
(TIF)
