# Supplementary figures and images for: The Transcriptional Activator LdtR from ‘Candidatus Liberibacter asiaticus’ Mediates Osmotic Stress Tolerance
Source: PLoS Pathog. 2014 Apr 24;10(4):e1004101. doi: 10.1371/journal.ppat.1004101 (PMC3999280; doi:10.1371/journal.ppat.1004101)

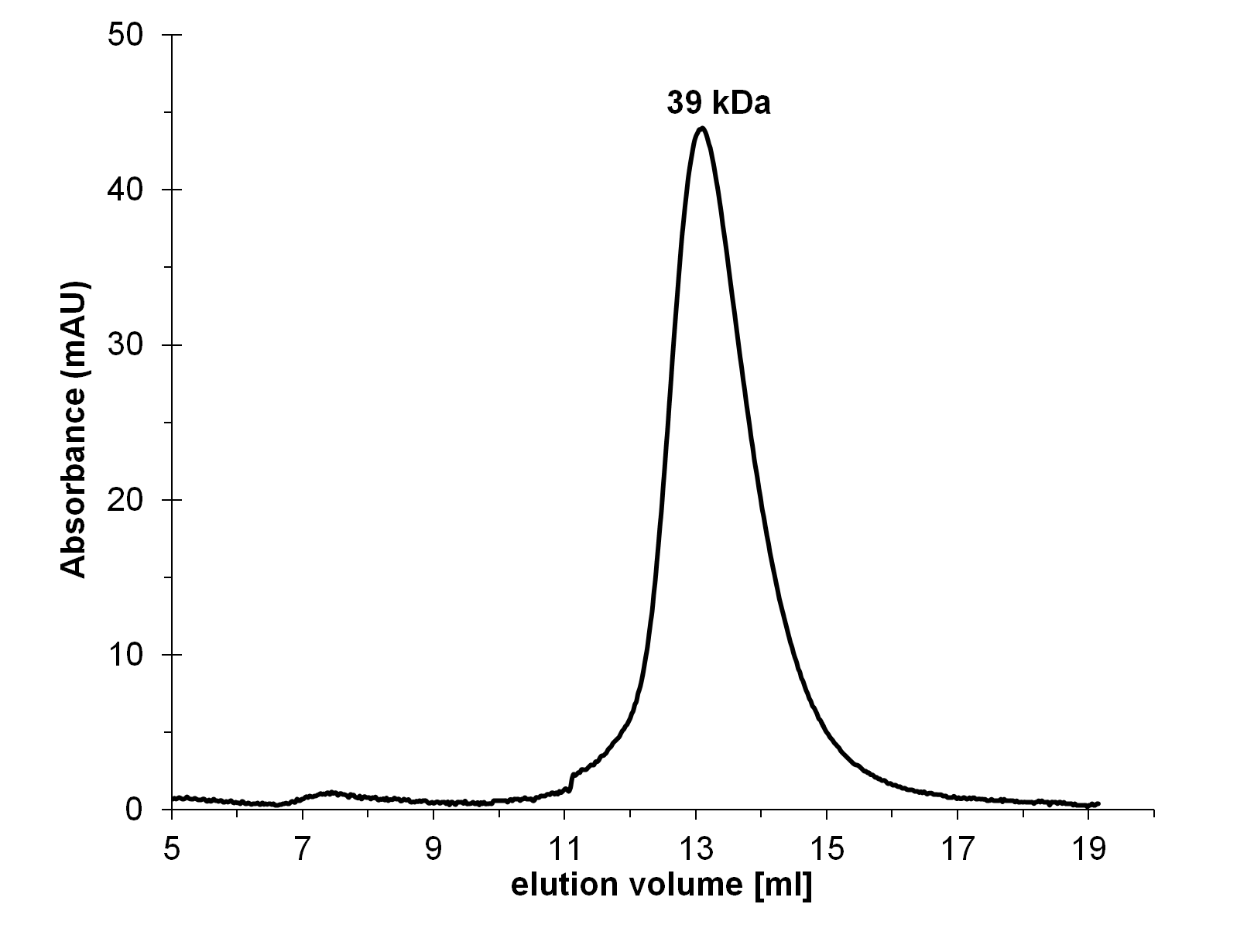

Supplement: Figure S1 — Determination of the oligomeric state of LdtRLas. Size-exclusion chromatography was performed using a Superose 12 column, as described in Materials and Methods section. (TIF) [file ppat.1004101.s001.tif]

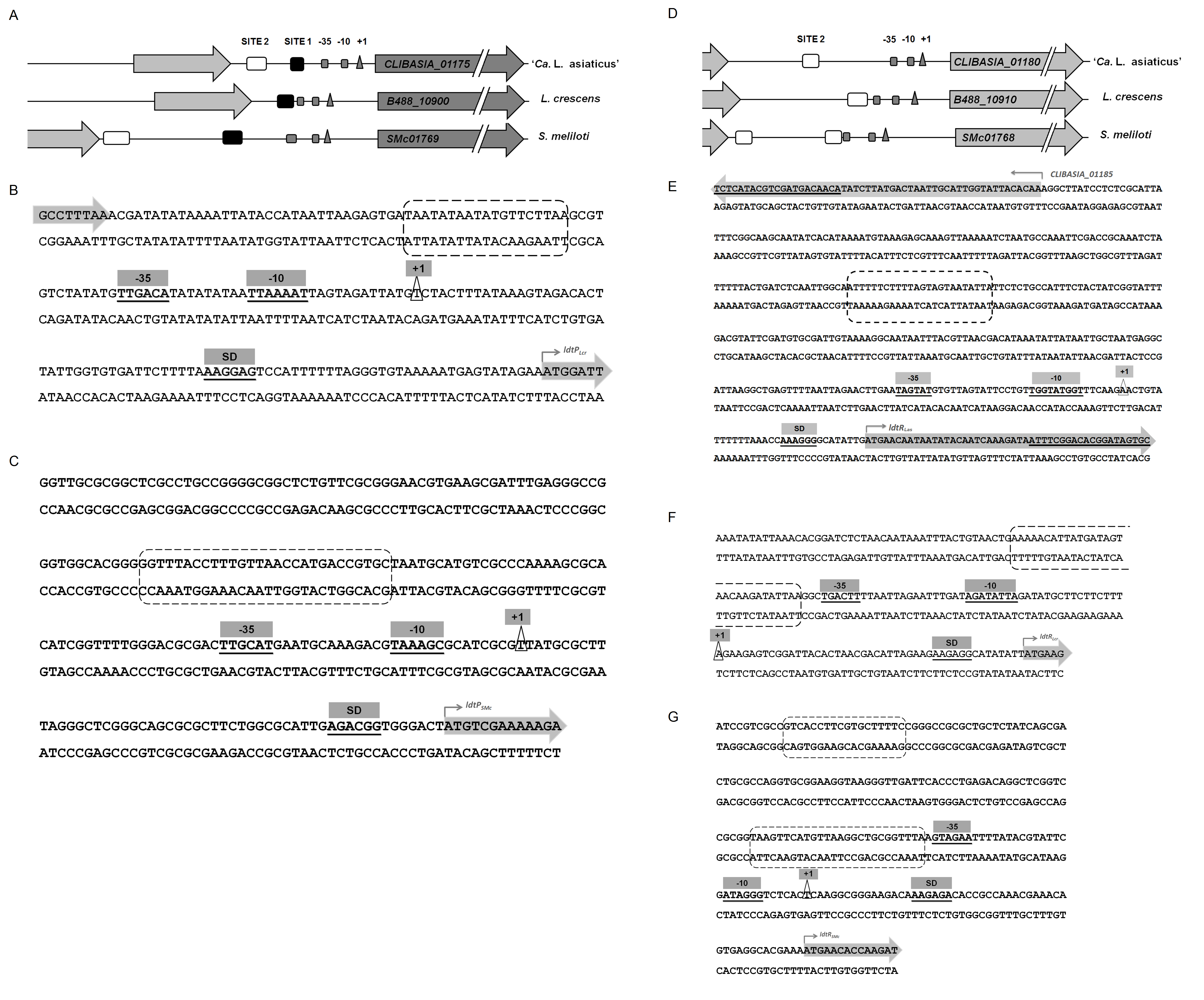

Supplement: Figure S2 — The location of LdtR binding sites in PldtR and PldtP is conserved upstream of the promoter elements in ‘ Ca. L. asiaticus’, L. crescens , and S. meliloti . (A) Graphical representation (to scale) of the LdtR binding sites and promoter elements of ldtP in ‘Ca. L. asiaticus’, L. crescens, and S. meliloti. Detailed characterization of PldtP in (B) L. crescens, or (C) S. meliloti. (D) Graphical representation (to scale) of the LdtR binding sites and promoter elements of ldtR in ‘Ca. L. asiaticus’, L. crescens, and S. meliloti. Detailed characterization of PldtR in (E) ‘Ca. L. asiaticus’, (F) L. crescens, or (G) S. meliloti. The experimentally determined transcription start site (+1) of ldtP and ldtR in ‘Ca. L. asiaticus’ and L. crescens, as well as the predicted transcription start site in S. meliloti are depicted in a triangle. The −10 and −35 boxes, as well as the Shine-Dalgarno sequence (SD) are underlined and highlighted in gray boxes. The putative binding sites for LdtR are identified by dashed boxes. (TIF) [file ppat.1004101.s002.tif]

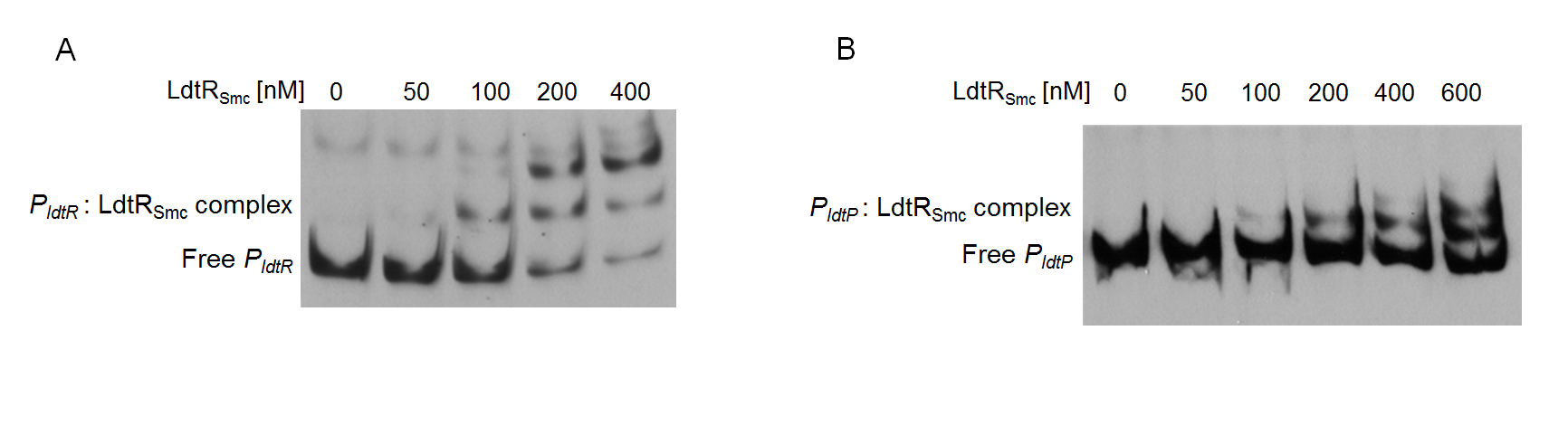

Supplement: Figure S3 — LdtRSmc binds to PldtR and PldtP of S. meliloti . EMSAs were conducted on (A) PldtR or (B) PldtP probes with increasing concentrations of LdtRSmc, as indicated on top of each panel. No protein was added to the first lane. (TIF) [file ppat.1004101.s003.tif]

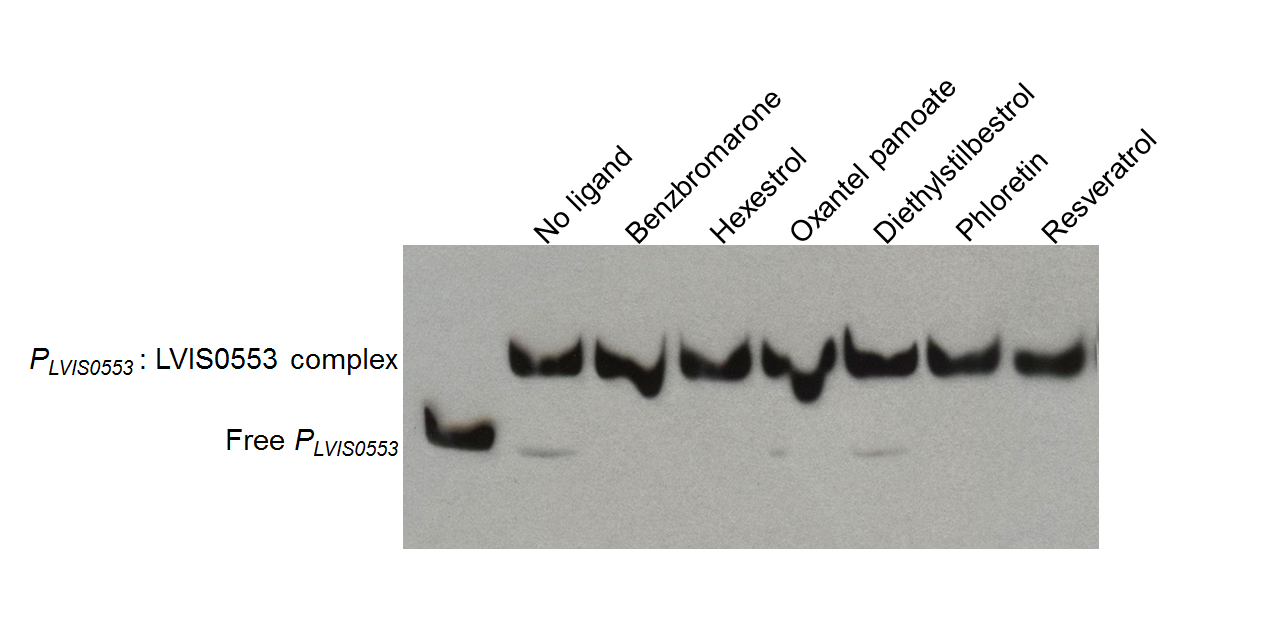

Supplement: Figure S5 — PLVIS0553: LVIS0553 interaction is not affected by LdtRLas ligands. EMSAs were conducted in the presence of 200 µM benzbromarone, hexestrol, oxantel pamoate, diethylstilbestrol, phloretin or resveratrol. The concentration of LVIS0553 was maintained at 20 nM. No protein was added to the first lane. (TIF) [file ppat.1004101.s005.tif]

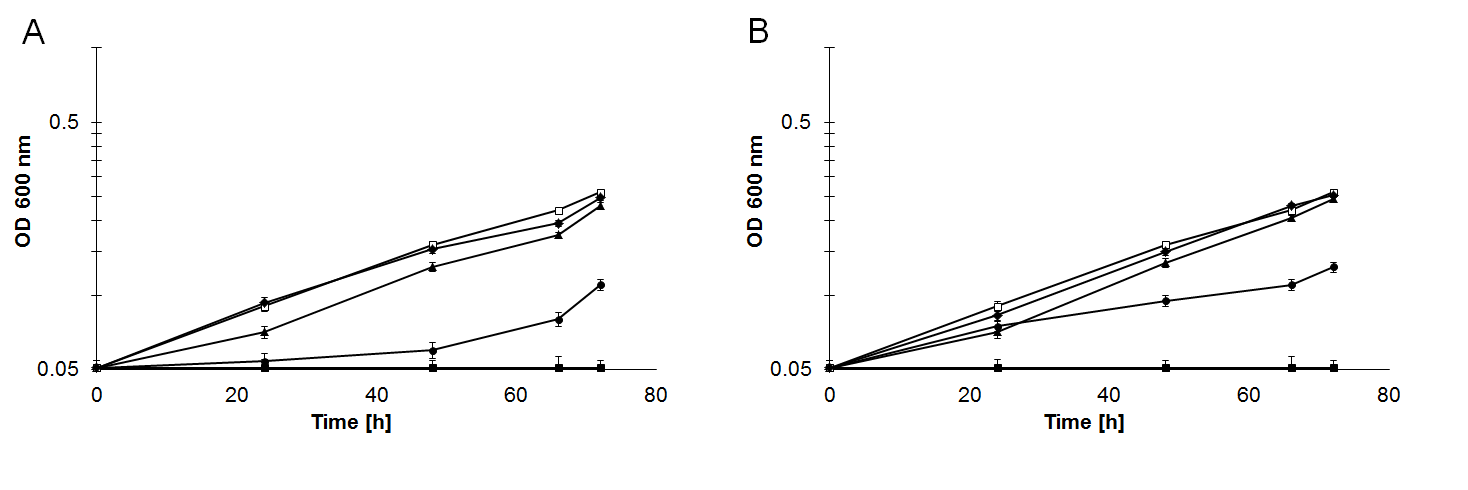

Supplement: Figure S6 — Growth of L. crescen s with increasing concentrations of NaCl or sucrose. In A, sucrose was added at 0 (empty square), 100 (diamond), 200 (triangle), 400 (circle) or 600 (filled square) mM. In B, NaCl was added at 0 (empty square), 100 (diamond), 150 (triangle), 200 (circle) or 400 (filled square). The growth curves were performed in triplicates. (TIF) [file ppat.1004101.s006.tif]
